# Preoperative health-related quality of life across four degenerative lumbar conditions in a multi-ethnic Southeast Asian surgical cohort: a cross-sectional SF-36 analysis

**DOI:** 10.1007/s11136-026-04336-3

**Published:** 2026-07-16

**Authors:** Yu Heng Yeow, Siew Tiang Lau, Xun Li, Calvin Wei Jie Chern, Xin Zhang, Alex Quok An Teo, Hwee Weng Dennis Hey, Ling Jie Cheng

**Affiliations:** 1 Alice Lee Centre for Nursing Studies, Yong Loo Lin School of Medicine, National University of Singapore, Singapore, Singapore; 2 School of Engineering, Computing, and Mathematics, Oxford Brookes University, Oxford, UK; 3 Saw Swee Hock School of Public Health, National University of Singapore, Singapore, Singapore; 4 Department of Orthopaedic Surgery, National University Hospital, Singapore, Singapore; 5 Department of Orthopaedic Surgery, Yong Loo Lin School of Medicine, National University of Singapore, Singapore, Singapore; 6 National Perinatal Epidemiology Unit, Nuffield Department of Women’s & Reproductive Health, University of Oxford, Oxford, UK

**Keywords:** Health-related quality of life, SF-36, Degenerative lumbar spine, Patient-reported outcome measures, Spinal stenosis, Asian population norms

## Abstract

**Purpose:**

The evidence base for preoperative health-related quality of life (HRQoL) in degenerative lumbar conditions derives predominantly from Western populations. This study characterised HRQoL across four degenerative lumbar conditions in a multi-ethnic Southeast Asian surgical cohort and identified factors associated with physical and mental health at composite and domain levels.

**Methods:**

Cross-sectional analysis of 1173 preoperative patients from a Singaporean spine surgery registry (2017–2022). SF-36 norm-based T-scores were computed for eight domains and two component summaries. Diagnosis-stratified stepwise multiple regression identified factors associated with the Physical Component Summary (PCS), Mental Component Summary (MCS), and domain scores. LASSO regression served as a sensitivity analysis.

**Results:**

Mean PCS was 35.6 (SD 8.9), 1.4 SD below the US normative mean, whereas MCS was 50.3 (SD 9.7), near normative levels. Physical functioning and role-physical were the most impaired domains. Scores differed across the four diagnoses on six of the ten SF-36 outcomes (one-way ANOVA, all *p* < 0.05; corroborated by Kruskal–Wallis tests), although effect sizes were small (eta-squared ≤ 0.02). Functional disability showed the strongest association with PCS across all four conditions (β range: − 0.32 to − 0.28; adjusted R^2^ = 0.48–0.52). MCS models explained limited variance (adjusted R^2^ = 0.09–0.22). At the domain level, physical functioning had the highest adjusted R^2^ (0.57–0.67), exceeding even PCS. Indian ethnicity was independently associated with lower MCS in two conditions. LASSO sensitivity analyses corroborated the stepwise findings.

**Conclusion:**

Functional disability was most strongly associated with physical HRQoL, channelled through physical functioning limitations. Conventional variables explained limited mental HRQoL variance, suggesting unmeasured psychological and cultural factors play a substantial role. While functional disability’s primacy was consistent with Western evidence, secondary patterns diverged from Western cohorts. These findings underscore the need to incorporate psychological and culturally adapted measures into preoperative assessment.

**Supplementary Information:**

The online version contains supplementary material available at https://doi.org/10.1007/s11136-026-04336-3.

## Abstract (plain language summary)

Most existing evidence on quality of life prior to spine surgery is derived from studies conducted in Western populations. In this study, we examined 1173 patients in Singapore awaiting surgery for four common spinal conditions. Using the SF-36 health questionnaire, we found that patients’ physical health scores were substantially lower than those of the general population, while mental health scores were relatively preserved. Difficulty in performing everyday activities was the factor most strongly associated with poorer physical health, rather than pain severity, body mass index, or comorbidities. In contrast, mental health was more difficult to explain, with commonly measured clinical factors accounting for only a small proportion of the observed variation. We also observed that patients of Indian ethnicity reported lower mental wellbeing compared to those of Chinese ethnicity. These findings suggest that determinants of quality of life may differ between Asian and Western populations and highlight the need to give greater consideration to psychological and cultural factors in the care of patients undergoing spine surgery.

**Supplementary Information:**

The online version contains supplementary material available at https://doi.org/10.1007/s11136-026-04336-3.

## Introduction

Degenerative lumbar conditions are the principal cause of chronic lower back pain, the leading cause of years lived with disability globally [1]. The economic burden is correspondingly vast: direct healthcare expenditure was estimated at US$47 billion in 2019, rising to US$216 billion when indirect losses were included [2, 3]. With failure of non-operative treatment, surgery is often pursued with common indications including spinal stenosis, spondylolisthesis, lumbar disc herniation (LDH), and degenerative disc disease (DDD) [4]. Characterising preoperative quality of life furnishes the baseline against which surgical benefit is judged and informs shared decision-making. HRQoL is an important outcome in these conditions because, beyond pain and functional impairment, it captures the broader consequences for physical functioning, mental wellbeing, social participation, and daily role performance, providing a patient-centred baseline that informs preoperative assessment, treatment decision-making, and the evaluation of postoperative outcomes.

Patient-reported outcome measures (PROMs) capture subjective dimensions of health that physiological measurement cannot detect [5, 6]. The Short Form-36 Health Survey (SF-36) is particularly well-suited to this purpose [7]. Its eight-domain architecture and two component summaries offer substantially greater discriminative capacity than the EQ-5D, particularly at the mild-to-moderate end of the severity spectrum where ceiling effects are problematic [8, 9].

Two complementary theoretical frameworks guide interpretation of factors associated with HRQoL. The Wilson and Cleary model [10], as revised by Ferrans and colleagues [11], posits a conceptual pathway from biological variables through symptom status, functional status, and general health perceptions to overall quality of life, with each level moderated by individual and environmental characteristics. Andersen’s Behavioural Model [12] provides a complementary taxonomy of predisposing, enabling, and need factors. Neither framework asserts strict causality; both are particularly useful in cross-cultural research where factor salience may shift with societal context.

The extant evidence base for preoperative HRQoL in degenerative lumbar conditions is disproportionately Western. Large registry studies, notably the Spine Patient Outcomes Research Trial (SPORT) [13], the Michigan Spine Surgery Improvement Collaborative (MSSIC) [14], the Swedish Spine Register [15], and the Norwegian Registry for Spine Surgery (NORspine) [16], have consistently linked higher BMI, female sex, functional disability, and psychological distress with worse HRQoL, but draw from predominantly Western populations. Asian contributions are sparse and geographically narrow: Japanese studies have examined HRQoL in lumbar spinal stenosis alone [17, 18], whilst a single Chinese study focused on paraspinal muscle correlates [19]. A recent Japanese cohort further documented persistent HRQoL decrements in untreated symptomatic lumbar spinal stenosis [20].

Singapore’s multi-ethnic population allows examination of ethnic variation within a shared, heavily subsidised healthcare system. Although individual conditions have been examined separately, few studies have directly compared preoperative HRQoL across multiple degenerative lumbar conditions within a single cohort, and none have done so in a multi-ethnic Asian surgical population. Such direct comparison is needed to establish whether HRQoL burden differs by underlying pathology or instead reflects a shared preoperative profile. This study therefore aimed to (1) characterise the preoperative HRQoL of adults with spinal stenosis, spondylolisthesis, LDH, or DDD using the eight domains and two component summary scores of the SF-36; and (2) identify factors associated with the PCS, MCS, and the eight domain scores, stratified by diagnosis.

## Methods

This study is reported in accordance with the Strengthening the Reporting of Observational Studies in Epidemiology (STROBE) guidelines for cross-sectional studies [21].

### Ethics

This study was conducted in accordance with the Declaration of Helsinki. Exemption from full review was granted by the local institutional review board prior to the commencement of the study, as the study involved analysis of anonymised registry data not meeting the institutional definition of human subject research.

### Design, setting, and participants

This cross-sectional analysis used preoperative data from a single-centre prospective spine registry at a tertiary-referral university spine centre. The registry enrols adults with degenerative lumbar conditions undergoing surgery and collects preoperative PROMs and clinical data. The target population was adults with degenerative lumbar conditions who proceeded to surgery; the registry captures consecutive surgical candidates at this centre and is therefore intended to represent this surgical population rather than the broader population managed conservatively. All consecutive patients diagnosed with spinal stenosis, spondylolisthesis, LDH, or DDD, who were enrolled between January 2017 and July 2022 were eligible for inclusion. The 2017–2022 window was chosen for two reasons. First, this analysis forms part of a broader programme of registry-based research developing a patient-reported outcome-based decision support tool [22, 23], and the same 2017–2022 extraction window was applied across the programme, in consultation with the treating clinicians, to provide a consistent and contemporary surgical cohort. Second, from 2017 the collection of patient-reported outcome measures was consistently embedded in the routine preoperative workflow and undertaken by trained Spine Centre staff, giving near-universal completion, so that preoperative data captured from this point are the most complete and consistent. The registry has been in continuous operation since 2007 and remains active; the window was therefore defined by data consistency and study scope rather than by any cessation of recruitment. Eligibility required adults with a confirmed clinical diagnosis of lumbar spinal stenosis, lumbar disc herniation, lumbar spondylolisthesis, or degenerative disc disease who were scheduled for lumbar spine surgery and had a complete baseline SF-36. Patients with previous lumbar spine surgery or non-degenerative spinal pathology (for example, tumour, infection, or trauma) were excluded, whereas no patient was excluded on the basis of comorbidity or cognitive impairment; consequently, the cohort reflects the full spectrum of degenerative surgical candidates rather than a comorbidity-restricted subset. All patients subsequently underwent lumbar spine surgery. Preoperative data for this study were extracted from the registry and collected using the Research Electronic Data Capture (REDCap) electronic data capture platform hosted locally within the hospital [24, 25].

### Outcome measures

The SF-36 yields eight domain scores and two component summary scores (Physical Component Summary [PCS] and Mental Component Summary [MCS]) [7, 26]. Item responses were transformed into norm-based T-scores using 1998 US population norms (mean 50, SD 10) [27, 28]. US norms were used in preference to Singaporean norms because the SF-36 component summary scores (PCS and MCS) are derived from factor-score coefficients estimated in the US normative sample, ensuring valid composite scoring and international comparability; this choice and its implications are considered further in the Limitations. The bodily pain domain was scored using the orthopaedic-specific algorithm described by Laucis and colleagues [29]. The SF-36 has been validated for use in Singapore’s multiethnic population [30].

Functional disability was assessed using the Oswestry Disability Index (ODI) on a 0–100 scale, with higher scores indicating greater disability. Pain severity was recorded on a 0 to 100 numerical rating scale [31].

### Covariates

Sociodemographic variables included age (21–44, 45–64, ≥ 65 years [32]), sex, ethnicity (Chinese, Indian, Malay, Other), educational attainment (Primary, Secondary, College/Diploma, University and above), smoking history (non-smoker, ex-smoker, current smoker), and employment status (employed versus not employed, the latter comprising retirees, homemakers, students, and unemployed). Clinical variables comprised body mass index (BMI; categorised using Southeast Asian thresholds: normal ≤ 22.9, overweight 23.0–27.4, obese ≥ 27.5 kg/m^2^ [33]), comorbidity (dichotomised as none versus one or more), accident history, workers’ compensation status, and level of spine pathology (L4/5, L4/5 and L5/S1, L5/S1, mixed (three or more spinal levels), others). These variables were selected a priori and organised using the two guiding frameworks. Following Andersen’s Behavioural Model, we specified predisposing factors (age, sex, ethnicity, and education), enabling factors (employment and workers’ compensation status), and need factors (functional disability, pain severity, comorbidity, and spinal pathology). Consistent with the Wilson and Cleary model, these variables span the biological, symptom, and functional levels positioned proximal to general health perceptions and overall quality of life, together with the individual and environmental characteristics that the model treats as moderators; this mapping, rather than statistical association alone, guided their inclusion.

### Sample size

No formal sample size calculation was performed. The total sample (n = 1173) and smallest subgroup (DDD, n = 202) exceeded 10 subjects per predictor variable [34, 35], thereby providing adequate statistical power for the regression analyses conducted.

### Statistical analysis

Continuous variables were summarised as means and standard deviations; categorical variables as frequencies and percentages. Distributional assumptions were examined using histograms, Q-Q plots, and Shapiro–Wilk tests [36]. Univariate associations between each covariate and PCS or MCS were evaluated using independent-samples t-tests for binary variables, one-way analysis of variance for multi-category variables, and Pearson correlation for continuous variables [37], all stratified by diagnosis. Given the large sample, parametric methods were applied in reliance on the central limit theorem; the distributional and residual diagnostics described above were therefore used to confirm model adequacy rather than as a basis for selecting tests. Differences in each SF-36 score across the four diagnostic groups were compared using one-way analysis of variance, with the Kruskal–Wallis test as a non-parametric confirmation and the Tukey honestly significant difference test for post-hoc pairwise comparisons; eta-squared was reported to quantify the magnitude of any between-diagnosis differences.

For each diagnostic subgroup, stepwise multiple linear regression models were constructed for PCS and MCS, with a p-to-enter threshold of 0.05. All covariates were offered to each model simultaneously. Model selection was corroborated through backward elimination and forward selection, with information criteria adjudicating discrepancies [38, 39]. Variance inflation factors were inspected to exclude multicollinearity (threshold > 10) [40]. Regression coefficients are reported as unstandardised β with 95% confidence intervals. Adjusted R^2^ values are reported to quantify the proportion of variance in PCS and MCS accounted for by each final model. As a sensitivity analysis, least absolute shrinkage and selection operator (LASSO) regression with tenfold cross-validation was performed for each outcome and diagnostic subgroup; variables selected by LASSO were then refitted using ordinary least squares (OLS) to obtain unbiased β coefficients and 95% confidence intervals. Each β represents the expected difference in the SF-36 T-score for a one-unit increase in a continuous predictor, or relative to the reference category for a categorical predictor. For example, the ODI coefficient for PCS in stenosis (β =  − 0.31) indicates that each additional ODI point corresponds to a 0.31-point lower PCS, so a 10-point higher ODI corresponds to an approximately 3-point lower PCS (about one-third of a T-score standard deviation). For a categorical predictor, the coefficient for Indian ethnicity on MCS in stenosis (β =  − 4.06) indicates a mean MCS approximately 4 points lower than the Chinese reference group, after adjustment for other retained variables.

All analyses were performed in Stata version 18.0 [41]. A two-sided *p*-value < 0.05 was considered statistically significant. No adjustments were made for multiple comparisons; findings should be interpreted accordingly.

## Results

### Participant characteristics

Of 1,195 registry patients, 22 were excluded (1 missing SF-36, 21 aged under 21), leaving 1,173 for analysis. Patients aged under 21 years were excluded a priori for two reasons: the adopted adult age-group framework (21–44, 45–64, and 65 years and above) begins at 21 years, so retaining younger patients would have produced an incomplete age-group classification; and degenerative lumbar pathology presenting before adulthood differs in aetiology, natural history, and management from adult degenerative disease, making it conceptually distinct from the target population. This group was small (n = 21). A companion analysis of the same registry that adopted an age framework beginning at 18 years retained these patients; the present analysis therefore differs by this small number of cases, reflecting the differing age-band definitions rather than any difference in the underlying cohort. Two patients had incomplete covariate data; regression analyses used complete cases.

The sociodemographic and clinical characteristics of the 1173 patients are summarised in Table 1. Males constituted 51.2%. The LDH group was younger (mean age 43.8 years, SD 14.2 years) than the stenosis group (65.0, SD 10.5). Mean ODI was 42.2 (SD 20.5) and pain severity 56.1 (SD 29.5).

**Table 1:** Sociodemographic and Clinical Characteristics

Characteristic	Overall (N = 1,173)	Stenosis (N = 480)	Spondylolisthesis (N = 229)	LDH (N = 262)	DDD (N = 202)
N	1173	480	229	262	202
Age, years	58.9 (15.3)	65.0 (10.5)	62.8 (13.0)	43.8 (14.2)	59.3 (15.4)
BMI, kg/m^2^	26.3 (4.7)	26.4 (4.7)	26.6 (5.2)	25.7 (4.4)	26.6 (4.6)
ODI score	42.2 (20.5)	42.1 (20.2)	40.2 (18.6)	43.7 (22.5)	42.9 (20.6)
Pain score	56.1 (29.5)	54.6 (28.5)	54.1 (28.2)	59.7 (32.1)	57.1 (29.5)
*Sex, n (%)*					
Female	572 (48.8)	225 (46.9)	134 (58.5)	105 (40.1)	108 (53.5)
Male	601 (51.2)	255 (53.1)	95 (41.5)	157 (59.9)	94 (46.5)
*Age group, n (%)*					
21–44	224 (19.1)	20 (4.2)	21 (9.2)	144 (55.0)	39 (19.3)
45–64	443 (37.8)	187 (39.0)	87 (38.0)	93 (35.5)	76 (37.6)
≥ 65	506 (43.1)	273 (56.9)	121 (52.8)	25 (9.5)	87 (43.1)
*Race, n (%)*					
Chinese	827 (70.5)	360 (75.0)	176 (76.9)	153 (58.4)	138 (68.3)
Indian	100 (8.5)	34 (7.1)	14 (6.1)	37 (14.1)	15 (7.4)
Malay	141 (12.0)	57 (11.9)	24 (10.5)	39 (14.9)	21 (10.4)
Other	105 (9.0)	29 (6.0)	15 (6.6)	33 (12.6)	28 (13.9)
*Education, n (%)*					
Primary	234 (19.9)	126 (26.2)	57 (24.9)	22 (8.4)	29 (14.4)
Secondary	364 (31.0)	165 (34.4)	72 (31.4)	62 (23.7)	65 (32.2)
College/Diploma	302 (25.7)	109 (22.7)	61 (26.6)	69 (26.3)	63 (31.2)
University & above	273 (23.3)	80 (16.7)	39 (17.0)	109 (41.6)	45 (22.3)
*Employment status, n (%)*					
Not employed	576 (49.1)	296 (61.7)	121 (52.8)	61 (23.3)	98 (48.5)
Employed	597 (50.9)	184 (38.3)	108 (47.2)	201 (76.7)	104 (51.5)
*BMI category, n (%)*					
Normal	271 (23.1)	104 (21.7)	51 (22.3)	73 (27.9)	43 (21.3)
Overweight	480 (40.9)	201 (41.9)	97 (42.4)	104 (39.7)	78 (38.6)
Obese	422 (36.0)	175 (36.5)	81 (35.4)	85 (32.4)	81 (40.1)
*Comorbidity, n (%)*					
No comorbidity	358 (30.5)	83 (17.3)	61 (26.6)	155 (59.2)	59 (29.2)
≥ 1 comorbidity	815 (69.5)	397 (82.7)	168 (73.4)	107 (40.8)	143 (70.8)
*Smoking history, n (%)*					
Non-smoker	914 (77.9)	367 (76.5)	183 (79.9)	195 (74.4)	169 (83.7)
Ex-smoker	101 (8.6)	47 (9.8)	20 (8.7)	23 (8.8)	11 (5.4)
Smoker	158 (13.5)	66 (13.8)	26 (11.4)	44 (16.8)	22 (10.9)
*Accident history, n (%)*					
No	1110 (94.6)	461 (96.0)	214 (93.4)	244 (93.1)	191 (94.6)
Yes	63 (5.4)	19 (4.0)	15 (6.6)	18 (6.9)	11 (5.4)
*Work compensation, n (%)*					
No	1164 (99.2)	480 (100.0)	227 (99.1)	258 (98.5)	199 (98.5)
Yes	9 (0.8)	0 (0.0)	2 (0.9)	4 (1.5)	3 (1.5)
*Spine level, n (%) *^*a*^					
L4/5	429 (36.6)	164 (34.2)	83 (36.2)	116 (44.3)	66 (32.7)
L4/5 and L5/S1	127 (10.8)	57 (11.9)	29 (12.7)	14 (5.3)	27 (13.4)
L5/S1	195 (16.6)	23 (4.8)	31 (13.5)	105 (40.1)	36 (17.8)
Mixed	82 (7.0)	51 (10.6)	10 (4.4)	6 (2.3)	15 (7.4)
Others	339 (28.9)	184 (38.4)	76 (33.2)	21 (8.0)	58 (28.7)

Values are *n (%)* for categorical variables and *mean (SD)* for continuous variables. BMI categories are based on the Asian classification (WHO, 2004 [33]). Abbreviations: BMI, body mass index; DDD, degenerative disc disease; LDH, lumbar disc herniation; ODI, Oswestry Disability Index; SD, standard deviation. ^a^ Spinal level data were missing for one patient with stenosis

### Preoperative SF-36 profile

**Fig. 1:**
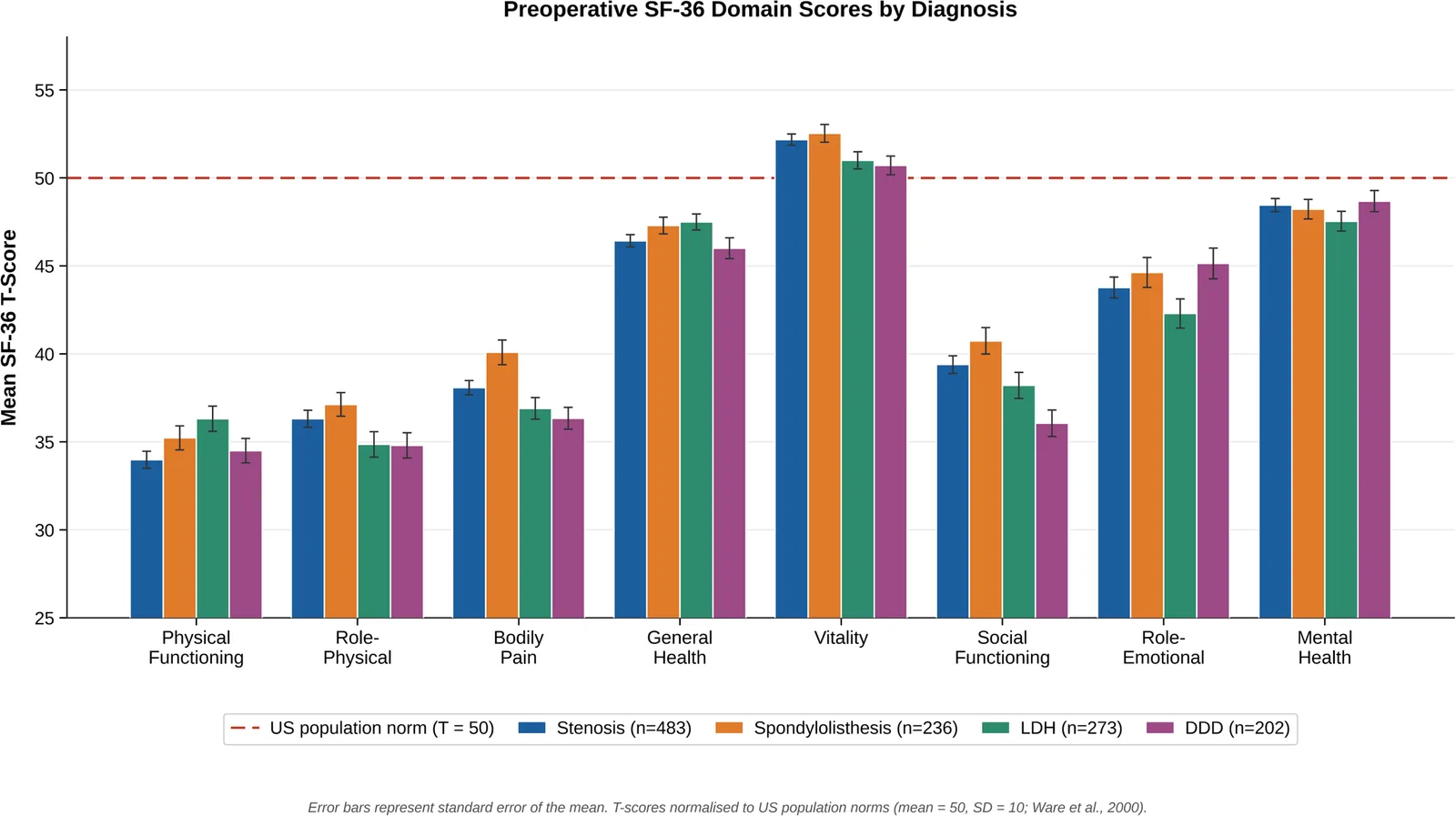
Preoperative SF-36 Domain Scores by Diagnosis. DDD = degenerative disc disease; LDH = lumbar disc herniation

**Fig. 2:**
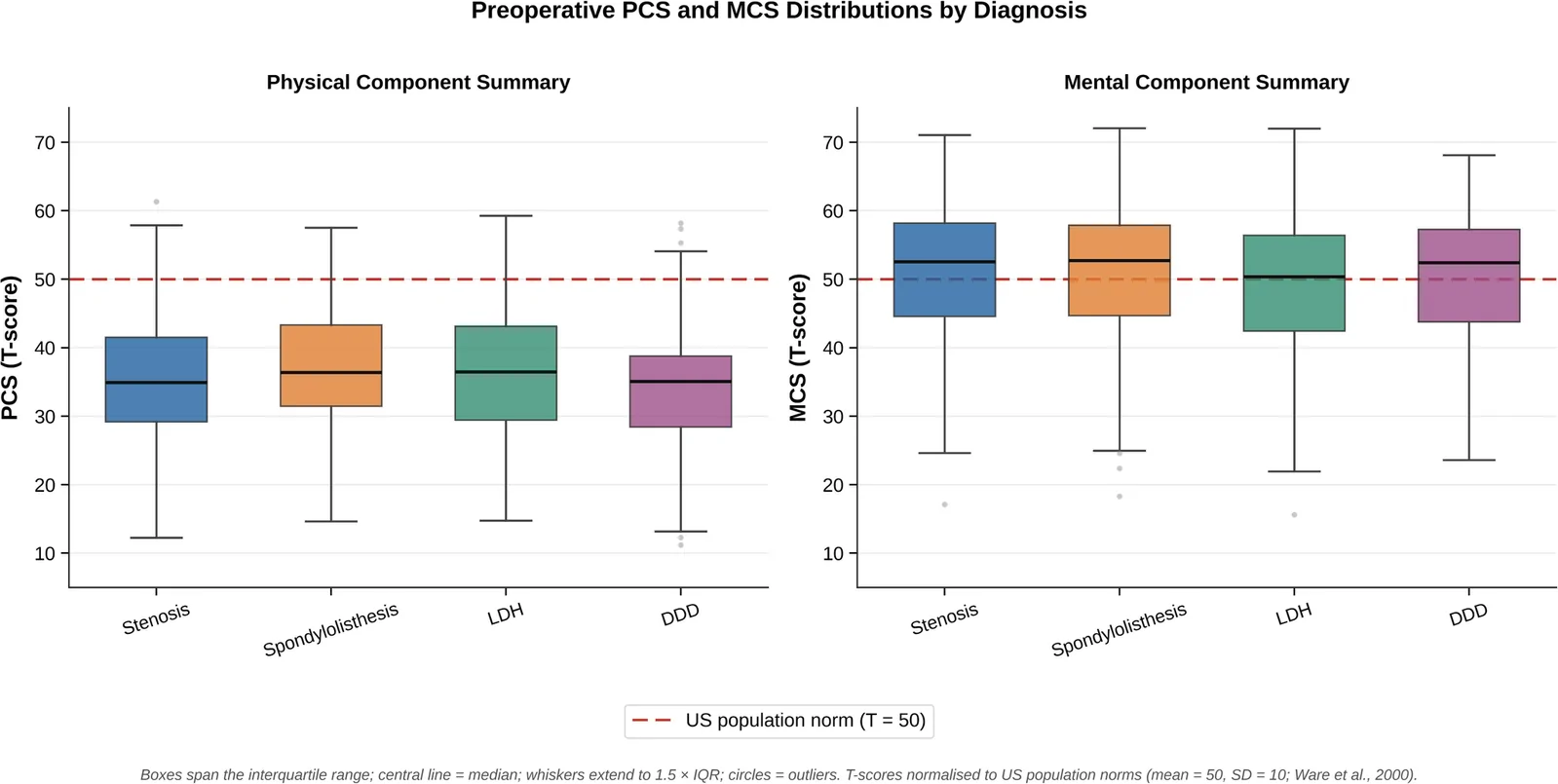
PCS and MCS Distributions by Diagnosis. DDD = degenerative disc disease; LDH = lumbar disc herniation; MCS = Mental Component Summary; PCS = Physical Component Summary

Mean PCS was 35.6 (SD 8.9), approximately 1.4 SD below the US normative mean [28] (Table 2). The mean MCS was 50.3 (SD 9.7), essentially at the normative mean [28]. Across the four diagnostic subgroups, PCS ranged from 34.1 (DDD) to 37.0 (spondylolisthesis), whereas MCS ranged from 48.8 (LDH) to 50.9 (spondylolisthesis). At the domain level, the lowest mean scores were observed for PF (34.7, SD 10.8) and RP (35.9, SD 10.8), approximately 1.5 and 1.4 standard deviations below the normative mean, respectively. The overall and diagnosis-specific SF-36 domain scores are presented in Table 2. The domain-level score profiles by diagnosis are displayed in Fig. 1, and the distributions of PCS and MCS by diagnosis are shown in Fig. 2. Formal comparison across the four diagnoses (Table 2) showed statistically significant differences for six of the ten SF-36 outcomes by one-way ANOVA, namely bodily pain (F[3,1169] = 7.81, *p* < 0.001), social functioning (F = 7.01, *p* < 0.001), PCS (F = 4.08, *p* = 0.007), vitality (F = 4.06, *p* = 0.007), role-physical (F = 3.38, *p* = 0.018), and MCS (F = 2.84, *p* = 0.037); physical functioning, general health, role-emotional, and mental health did not differ significantly (all *p* > 0.05). Kruskal–Wallis tests yielded concordant results. Despite reaching statistical significance, all effect sizes were small (eta-squared ≤ 0.02), indicating that between-diagnosis differences were modest relative to within-group variation. In Tukey post-hoc comparisons, the largest separations were on bodily pain, where spondylolisthesis exceeded DDD (mean difference 3.87, *p* < 0.001), LDH (3.57, *p* < 0.001), and stenosis (2.14, *p* = 0.028), and on social functioning, where DDD was lower than spondylolisthesis (4.67, *p* < 0.001) and stenosis (3.33, *p* = 0.003), and LDH was lower than spondylolisthesis (2.82, *p* = 0.032). Remaining significant pairwise differences were small (PCS: DDD lower than spondylolisthesis, 2.92, *p* = 0.004; MCS: LDH lower than stenosis, 2.02, *p* = 0.034).

**Table 2:** Preoperative SF-36 T-Scores by Diagnosis

SF-36 Domain	Overall (N = 1,173)	Stenosis (N = 480)	Spondylolisthesis (N = 229)	LDH (N = 262)	DDD (N = 202)	P value†
*Physical Domains*						
Physical Component Summary	35.6 (8.9)	35.4 (8.8)	37.0 (8.5)	35.8 (9.4)	34.1 (8.5)	0.007
Physical Functioning	34.7 (10.8)	33.9 (10.6)	35.1 (10.5)	35.8 (11.9)	34.5 (9.8)	0.116
Role-Physical	35.9 (10.8)	36.4 (10.6)	37.2 (10.3)	34.6 (12.0)	34.8 (10.2)	0.018
Bodily Pain	37.9 (9.6)	38.1 (9.0)	40.2 (10.8)	36.6 (9.9)	36.3 (8.8)	< 0.001
General Health	46.7 (7.7)	46.4 (7.6)	47.4 (7.2)	47.3 (7.5)	46.0 (8.5)	0.141
*Mental Domains*						
Mental Component Summary	50.3 (9.7)	50.8 (9.5)	50.9 (9.6)	48.8 (10.2)	50.5 (9.5)	0.037
Vitality	51.7 (7.5)	52.2 (7.1)	52.6 (7.6)	50.9 (8.0)	50.7 (7.6)	0.007
Social Functioning	38.7 (11.5)	39.4 (11.1)	40.7 (11.6)	37.9 (12.4)	36.1 (10.7)	< 0.001
Role-Emotional	43.8 (13.2)	43.8 (13.1)	44.5 (13.2)	42.2 (13.7)	45.1 (12.4)	0.079
Mental Health	48.2 (8.6)	48.5 (8.2)	48.3 (8.5)	47.4 (9.4)	48.7 (8.5)	0.357

Values are *mean (SD)*. T scores are normalised to US population norms (mean = 50, SD = 10; Ware et al., 2000 [28]). Higher scores indicate better health. Abbreviations: DDD, degenerative disc disease; LDH, lumbar disc herniation; SD, standard deviation. † P value from one-way ANOVA comparing the four diagnostic groups; differences were corroborated by Kruskal–Wallis tests, and all effect sizes were small (eta-squared ≤ 0.02). Post-hoc pairwise comparisons are reported in the main text

### Univariate associations

PCS showed strong negative correlations with ODI across all diagnostic subgroups (Pearson r range − 0.72 to − 0.69) and was also negatively correlated with pain severity (β range − 0.13 to − 0.12 per unit increase in pain score). Both metrics are presented in Table 3.

**Table 3:** Univariate Linear Regression: PCS and MCS by Diagnosis

Variable	Category	Stenosis (N = 480)		Spondylolisthesis (N = 229)		LDH (N = 262)		DDD (N = 202)	
		Mean (SD)	β (95% CI)	Mean (SD)	β (95% CI)	Mean (SD)	β (95% CI)	Mean (SD)	β (95% CI)
*Physical Component Summary (PCS)*									
*Sex*									
	Female	34.0 (8.2)	Ref	36.6 (7.9)	Ref	35.0 (9.6)	Ref	33.8 (7.6)	Ref
	Male	36.6 (9.1)	2.62 (1.06, 4.18)*	37.6 (9.4)	0.93 (−1.33, 3.19)	36.4 (9.2)	1.35 (−0.98, 3.68)	34.5 (9.4)	0.68 (−1.69, 3.05)
* Age group*									
	21–44	31.8 (8.7)	Ref	41.5 (8.7)	Ref	36.0 (9.0)	Ref	33.2 (9.4)	Ref
	45–64	36.6 (8.8)	4.78 (0.76, 8.81)*	36.6 (8.5)	−4.95 (−9.00, −0.90)*	35.4 (9.5)	−0.64 (−3.11, 1.83)	34.5 (9.2)	1.36 (−1.95, 4.67)
	≥ 65	34.8 (8.6)	3.01 (−0.96, 6.97)	36.5 (8.4)	−5.00 (−8.94, −1.06)*	36.4 (11.1)	0.35 (−3.67, 4.38)	34.2 (7.4)	0.99 (−2.25, 4.23)
* Race*									
	Chinese	35.9 (8.6)	Ref	37.4 (8.6)	Ref	36.5 (9.7)	Ref	35.2 (8.1)	Ref
	Indian	36.2 (9.6)	0.35 (−2.72, 3.42)	37.0 (7.0)	−0.44 (−5.09, 4.22)	35.4 (8.2)	−1.14 (−4.53, 2.25)	29.3 (6.8)	−5.86 (−10.31, −1.41)*
	Malay	32.4 (8.3)	−3.50 (−5.95, −1.06)*	33.4 (8.8)	−4.05 (−7.70, −0.41)*	33.7 (8.8)	−2.81 (−6.14, 0.51)	29.9 (7.7)	−5.23 (−9.06, −1.40)*
	Other	34.1 (9.9)	−1.79 (−5.10, 1.51)	38.4 (7.9)	1.01 (−3.50, 5.52)	35.6 (9.8)	−0.97 (−4.53, 2.58)	34.6 (10.3)	−0.57 (−3.97, 2.82)
* Education*									
	Primary	31.5 (7.4)	Ref	35.5 (8.5)	Ref	33.1 (9.2)	Ref	30.7 (7.5)	Ref
	Secondary	35.7 (8.4)	4.16 (2.20, 6.12)*	35.2 (8.5)	−0.33 (−3.26, 2.60)	33.9 (8.7)	0.87 (−3.66, 5.40)	34.2 (7.9)	3.57 (−0.13, 7.27)
	College/Diploma	37.1 (8.9)	5.58 (3.41, 7.74)*	39.6 (8.3)	4.06 (1.02, 7.11)*	35.2 (9.9)	2.11 (−2.36, 6.58)	34.1 (8.8)	3.47 (−0.25, 7.19)
	University & above	38.5 (9.4)	7.00 (4.63, 9.36)*	38.5 (8.1)	3.00 (−0.44, 6.43)	37.9 (9.2)	4.82 (0.55, 9.09)*	36.1 (9.1)	5.46 (1.51, 9.41)*
* Employment*									
	Not employed	34.1 (8.9)	Ref	35.8 (8.4)	Ref	33.9 (9.3)	Ref	32.0 (8.0)	Ref
	Employed	37.5 (8.2)	3.38 (1.79, 4.97)*	38.4 (8.6)	2.64 (0.43, 4.85)*	36.4 (9.4)	2.58 (−0.11, 5.28)	36.1 (8.5)	4.15 (1.86, 6.44)*
* BMI category*									
	Normal	36.9 (8.6)	Ref	39.5 (7.7)	Ref	34.9 (9.6)	Ref	33.7 (8.2)	Ref
	Overweight	35.7 (8.4)	−1.27 (−3.34, 0.80)	37.0 (9.0)	−2.48 (−5.36, 0.40)	36.6 (9.0)	1.66 (−1.17, 4.49)	36.7 (7.7)	3.04 (−0.05, 6.13)
	Obese	34.1 (9.1)	−2.81 (−4.93, −0.69)*	35.5 (8.2)	−3.97 (−6.94, −0.99)*	35.7 (9.7)	0.74 (−2.22, 3.70)	31.8 (8.8)	−1.82 (−4.89, 1.25)
* Comorbidity*									
	No comorbidity	36.2 (9.5)	Ref	38.8 (9.1)	Ref	36.1 (9.2)	Ref	36.0 (9.5)	Ref
	≥ 1 comorbidity	35.2 (8.6)	−0.95 (−3.03, 1.13)	36.4 (8.3)	−2.48 (−4.98, 0.02)	35.5 (9.7)	−0.53 (−2.86, 1.80)	33.3 (8.0)	−2.70 (−5.27, −0.13)*
* Smoking history*									
	Non-smoker	35.7 (8.9)	Ref	37.2 (8.5)	Ref	36.2 (9.2)	Ref	34.6 (8.0)	Ref
	Ex-smoker	34.5 (8.1)	−1.21 (−3.88, 1.46)	36.6 (9.0)	−0.56 (−4.54, 3.42)	38.1 (9.5)	1.95 (−2.11, 6.01)	34.3 (9.3)	−0.21 (−5.40, 4.97)
	Smoker	34.2 (8.4)	−1.49 (−3.80, 0.81)	36.4 (8.6)	−0.71 (−4.25, 2.83)	33.2 (9.7)	−2.94 (−6.02, 0.13)	30.6 (11.2)	−4.00 (−7.77, −0.22)*
* Accident*									
	No	35.6 (8.7)	Ref	37.0 (8.4)	Ref	35.9 (9.5)	Ref	34.3 (8.5)	Ref
	Yes	29.9 (7.9)	−5.70 (−9.71, −1.70)*	37.1 (11.2)	0.11 (−4.39, 4.62)	34.8 (8.5)	−1.10 (−5.63, 3.43)	30.6 (8.5)	−3.74 (−8.92, 1.44)
* Work compensation*									
	No	35.4 (8.8)	Ref	36.9 (8.5)	Ref	35.8 (9.4)	Ref	34.2 (8.4)	Ref
	Yes	–	–	50.8 (7.2)	13.85 (2.00, 25.70)*	37.4 (9.1)	1.59 (−7.75, 10.94)	26.3 (9.7)	−7.89 (−17.60, 1.82)
* Spine level*									
	L4/5	35.9 (8.5)	Ref	36.2 (8.4)	Ref	36.3 (10.1)	Ref	34.7 (8.5)	Ref
	L4/5 and L5/S1	36.5 (8.7)	0.63 (−2.02, 3.28)	37.4 (9.5)	1.22 (−2.44, 4.87)	39.1 (8.9)	2.82 (−2.43, 8.07)	33.6 (6.8)	−1.11 (−4.97, 2.75)
	L5/S1	36.1 (8.4)	0.24 (−3.60, 4.07)	38.1 (9.6)	1.93 (−1.64, 5.49)	35.1 (8.7)	−1.12 (−3.62, 1.38)	34.3 (10.3)	−0.40 (−3.89, 3.10)
	Mixed	33.4 (8.8)	−2.45 (−5.21, 0.31)	36.7 (10.1)	0.53 (−5.13, 6.20)	34.3 (4.3)	−1.98 (−9.75, 5.79)	32.7 (6.0)	−2.03 (−6.86, 2.80)
	Others	35.0 (9.0)	−0.83 (−2.68, 1.02)	37.4 (7.8)	1.29 (−1.40, 3.98)	35.4 (10.3)	−0.83 (−5.23, 3.57)	33.9 (8.7)	−0.78 (−3.82, 2.26)
* Continuous variables*									
ODI score	*r*	−0.723*		−0.713*		−0.693*		−0.689*	
	*β (95% CI)*		−0.31 (−0.34, −0.29)*		−0.33 (−0.37, −0.29)*		−0.29 (−0.33, −0.25)*		−0.28 (−0.33, −0.24)*
Pain score	*r*	−0.405*		−0.423*		−0.404*		−0.403*	
	*β (95% CI)*		−0.12 (−0.15, −0.10)*		−0.13 (−0.16, −0.09)*		−0.12 (−0.15, −0.09)*		−0.12 (−0.15, −0.08)*
*Mental Component Summary (MCS)*									
*Sex*									
	Female	49.8 (9.7)	Ref	49.6 (10.0)	Ref	48.6 (11.3)	Ref	49.3 (10.0)	Ref
	Male	51.7 (9.3)	1.97 (0.27, 3.68)*	52.6 (8.7)	3.03 (0.53, 5.54)*	48.9 (9.4)	0.28 (−2.26, 2.81)	51.9 (8.7)	2.59 (−0.03, 5.21)
* Age group*									
	21–44	48.7 (8.4)	Ref	49.3 (9.3)	Ref	49.2 (10.5)	Ref	48.5 (9.7)	Ref
	45–64	50.1 (9.1)	1.36 (−3.04, 5.76)	50.1 (8.6)	0.84 (−3.76, 5.43)	48.7 (9.5)	−0.53 (−3.20, 2.15)	50.3 (9.6)	1.85 (−1.82, 5.53)
	≥ 65	51.5 (9.8)	2.72 (−1.61, 7.05)	51.7 (10.3)	2.38 (−2.09, 6.84)	47.0 (11.0)	−2.24 (−6.60, 2.12)	51.6 (9.2)	3.14 (−0.45, 6.74)
* Race*									
	Chinese	51.5 (9.2)	Ref	51.4 (9.7)	Ref	49.1 (10.5)	Ref	51.0 (9.4)	Ref
	Indian	46.7 (9.8)	−4.80 (−8.13, −1.46)*	45.2 (8.2)	−6.21 (−11.42, −0.99)*	48.1 (9.5)	−0.99 (−4.68, 2.70)	46.7 (12.4)	−4.31 (−9.38, 0.77)
	Malay	49.4 (9.5)	−2.06 (−4.71, 0.59)	50.4 (10.0)	−1.00 (−5.09, 3.08)	47.4 (10.5)	−1.69 (−5.30, 1.92)	49.1 (8.5)	−1.90 (−6.27, 2.47)
	Other	50.7 (11.9)	−0.75 (−4.34, 2.83)	50.9 (7.7)	−0.54 (−5.59, 4.51)	49.7 (9.4)	0.55 (−3.31, 4.42)	51.3 (8.9)	0.33 (−3.54, 4.20)
*Education*									
	Primary	49.9 (9.8)	Ref	50.7 (8.4)	Ref	45.6 (11.4)	Ref	48.3 (10.9)	Ref
	Secondary	50.6 (9.6)	0.63 (−1.59, 2.84)	51.7 (10.5)	1.00 (−2.36, 4.35)	49.3 (9.0)	3.76 (−1.23, 8.74)	50.8 (9.8)	2.51 (−1.67, 6.70)
	College/Diploma	51.0 (9.6)	1.10 (−1.35, 3.55)	51.2 (8.4)	0.52 (−2.96, 4.01)	49.5 (9.6)	3.87 (−1.04, 8.79)	50.9 (8.5)	2.62 (−1.59, 6.82)
	University & above	52.4 (8.8)	2.49 (−0.18, 5.17)	48.9 (11.1)	−1.79 (−5.72, 2.15)	48.8 (10.9)	3.17 (−1.52, 7.87)	51.0 (9.5)	2.77 (−1.69, 7.23)
*Employment*									
	Not employed	51.0 (9.6)	Ref	51.5 (10.1)	Ref	46.6 (11.0)	Ref	50.0 (9.5)	Ref
	Employed	50.5 (9.5)	−0.50 (−2.25, 1.26)	50.1 (9.0)	−1.42 (−3.92, 1.08)	49.5 (9.9)	2.87 (−0.04, 5.79)	51.0 (9.5)	1.00 (−1.63, 3.64)
*BMI category*									
	Normal	48.9 (10.8)	Ref	49.1 (10.9)	Ref	47.0 (11.4)	Ref	51.5 (9.1)	Ref
	Overweight	51.6 (9.2)	2.69 (0.44, 4.95)*	51.4 (9.3)	2.28 (−0.98, 5.55)	49.0 (9.1)	2.01 (−1.05, 5.06)	52.1 (8.8)	0.61 (−2.90, 4.11)
	Obese	51.1 (9.0)	2.17 (−0.14, 4.48)	51.4 (9.1)	2.26 (−1.11, 5.64)	50.1 (10.3)	3.06 (−0.13, 6.25)	48.4 (10.0)	−3.09 (−6.58, 0.39)
*Comorbidity*									
	No comorbidity	52.3 (8.2)	Ref	49.3 (9.2)	Ref	49.1 (10.1)	Ref	50.7 (9.4)	Ref
	≥ 1 comorbidity	50.5 (9.8)	−1.76 (−4.01, 0.50)	51.4 (9.7)	2.09 (−0.72, 4.91)	48.4 (10.3)	−0.74 (−3.27, 1.78)	50.4 (9.5)	−0.31 (−3.21, 2.59)
*Smoking history*									
	Non-smoker	50.9 (9.2)	Ref	51.0 (9.8)	Ref	48.2 (10.3)	Ref	50.5 (9.8)	Ref
	Ex-smoker	53.3 (9.7)	2.42 (−0.47, 5.30)	50.8 (8.3)	−0.15 (−4.62, 4.31)	50.1 (10.9)	1.95 (−2.46, 6.37)	50.9 (8.5)	0.31 (−5.53, 6.15)
	Smoker	48.6 (10.7)	−2.29 (−4.78, 0.20)	50.4 (9.0)	−0.59 (−4.57, 3.38)	50.8 (9.3)	2.63 (−0.71, 5.98)	50.0 (8.0)	−0.53 (−4.78, 3.73)
*Accident*									
	No	50.8 (9.6)	Ref	51.0 (9.3)	Ref	48.6 (10.1)	Ref	50.4 (9.4)	Ref
	Yes	52.1 (8.4)	1.28 (−3.11, 5.66)	48.5 (13.4)	−2.50 (−7.55, 2.54)	51.1 (11.1)	2.46 (−2.44, 7.36)	52.1 (11.1)	1.67 (−4.14, 7.47)
*Work compensation*									
	No	50.8 (9.5)	Ref	50.9 (9.6)	Ref	48.8 (10.3)	Ref	50.5 (9.5)	Ref
	Yes	–	–	50.0 (3.7)	−0.88 (−14.32, 12.56)	51.5 (3.3)	2.77 (−7.36, 12.89)	50.7 (9.1)	0.19 (−10.71, 11.09)
*Spine level*									
	L4/5	50.0 (10.1)	Ref	51.1 (9.8)	Ref	49.5 (9.9)	Ref	50.9 (7.8)	Ref
	L4/5 and L5/S1	53.4 (9.3)	3.45 (0.58, 6.32)*	49.7 (8.7)	−1.43 (−5.47, 2.62)	49.2 (7.2)	−0.31 (−5.99, 5.37)	49.4 (12.1)	−1.48 (−5.78, 2.82)
	L5/S1	50.0 (7.3)	0.04 (−4.11, 4.20)	51.5 (9.1)	0.42 (−3.53, 4.37)	48.8 (10.8)	−0.66 (−3.37, 2.04)	49.8 (10.1)	−1.04 (−4.94, 2.86)
	Mixed	49.3 (10.1)	−0.70 (−3.69, 2.29)	43.6 (13.9)	−7.53 (−13.81, −1.25)*	47.4 (8.7)	−2.08 (−10.49, 6.32)	51.7 (7.9)	0.81 (−4.58, 6.19)
	Others	51.3 (9.0)	1.28 (−0.73, 3.28)	51.8 (9.0)	0.67 (−2.31, 3.64)	45.0 (10.6)	−4.51 (−9.27, 0.25)	50.7 (10.0)	−0.15 (−3.55, 3.24)
*Continuous variables*									
ODI score	*r*	−0.337*		−0.298*		−0.281*		−0.455*	
	*β (95% CI)*		−0.16 (−0.20, −0.12)*		−0.15 (−0.22, −0.09)*		−0.13 (−0.18, −0.07)*		−0.21 (−0.27, −0.15)*
Pain score	*r*	−0.221*		−0.195*		−0.255*		−0.355*	
	β (95% CI)		−0.07 (−0.10, −0.04)*		−0.07 (−0.11, −0.02)*		−0.08 (−0.12, −0.04)*		−0.11 (−0.16, −0.07)*

β = unstandardised regression coefficient (mean difference from reference for categorical; per-unit change for continuous). * *p* < 0.05. Ref = reference category. r = Pearson correlation coefficient. — = insufficient data. Abbreviations: DDD, degenerative disc disease; LDH, lumbar disc herniation; PCS, Physical Component Summary; MCS, Mental Component Summary; ODI, Oswestry Disability Index; BMI, body mass index; SD, standard deviation; CI, confidence interval

Higher educational attainment was associated with higher PCS in all four diagnostic subgroups, with the strongest and most consistent gradient observed in stenosis. Employment was associated with higher PCS in stenosis, spondylolisthesis, and DDD, but not LDH. Male sex was associated with higher PCS in stenosis only. Comorbidity was associated with lower PCS in DDD (β − 2.70, 95% CI − 5.27 to − 0.13), and current smoking was negatively associated with PCS in DDD (β − 4.00, 95% CI − 7.77 to − 0.22). BMI and accident history were non-significant across most groups.

MCS showed fewer associations. Correlations between ODI and MCS were small (DDD: r − 0.46; other conditions ranged from − 0.28 to − 0.34). Pain severity showed small negative associations with MCS in DDD (β − 0.11, 95% CI − 0.16 to − 0.07) and LDH (β − 0.08, 95% CI − 0.12 to − 0.04), with smaller associations in stenosis (β − 0.07, 95% CI − 0.10 to − 0.04) and spondylolisthesis (β − 0.07, 95% CI − 0.11 to − 0.02). Ethnicity showed differences in MCS in stenosis and spondylolisthesis, with Indian ethnicity associated with lower scores compared with Chinese ethnicity in both (stenosis: β−4.80, 95% CI−8.13 to−1.46; spondylolisthesis: β−6.66, 95% CI−11.42 to−0.99).

### Multivariable models

#### PCS

The diagnosis-stratified PCS models are presented in Table 4. ODI was retained in every model (β range − 0.32 to − 0.28; adjusted R^2^ 0.48–0.52). In stenosis and LDH, ODI was the sole retained variable. In spondylolisthesis, college/diploma education was positively associated with PCS (β = 2.14; 95% CI 0.37, 3.92). In the DDD model, comorbidity (β =  − 3.23; 95% CI: − 5.07, − 1.40), current smoker status (β =  − 3.08; 95% CI: − 5.74, − 0.41), and Malay ethnicity (β =  − 3.09; 95% CI: − 5.84, − 0.35) were also retained.

**Table 4:** Stepwise Multiple Linear Regression: PCS and MCS by Diagnosis

Variable	Stenosis		Spondylolisthesis		LDH		DDD	
	β (95% CI)	VIF	β (95% CI)	VIF	β (95% CI)	VIF	β (95% CI)	VIF
*Physical Component Summary (PCS)*								
	*N* = *479, Adj.R*^*2*^ = *0.5245*		*N* = *228, Adj.R*^*2*^ = *0.5167*		*N* = *262, Adj.R*^*2*^ = *0.4777*		*N* = *202, Adj.R*^*2*^ = *0.5113*	
ODI score	–0.31 (–0.34, –0.29)*	1.00	–0.32 (–0.36, –0.28)*	1.01	–0.29 (–0.33, –0.25)*	1.00	–0.28 (–0.32, –0.24)*	1.02
College/Diploma (ref: Primary)	–	–	2.14 (0.37, 3.92)*	1.01	–	–	–	–
≥ 1 comorbidity (ref: No comorbidity)	–	–	–	–	–	–	–3.23 (–5.07, –1.40)*	1.03
Smoker (ref: Non-smoker)	–	–	–	–	–	–	–3.08 (–5.74, –0.41)*	1.01
Malay (ref: Chinese)	–	–	–	–	–	–	–3.09 (–5.84, –0.35)*	1.03
*Mental Component Summary (MCS)*								
	*N* = *479, Adj.R*^*2*^ = *0.1369*		*N* = *228, Adj.R*^*2*^ = *0.1459*		*N* = *262, Adj.R*^*2*^ = *0.0863*		*N* = *202, Adj.R*^*2*^ = *0.2170*	
ODI score	–0.17 (–0.21, –0.13)*	1.03	–0.15 (–0.21, –0.09)*	1.00	–0.13 (–0.19, –0.08)*	1.01	–0.17 (–0.24, –0.10)*	1.39
Pain score	–	–	–	–	–	–	–0.05 (–0.10, –0.00)*	1.39
Indian (ref: Chinese)	–4.06 (–7.18, –0.94)*	1.01	–6.66 (–11.48, –1.83)*	1.00	–	–	–	–
Male (ref: Female)	–	–	3.11 (0.76, 5.46)*	1.00	–	–	–	–
≥ 65 years (ref: 21–44)	1.94 (0.31, 3.58)*	1.04	–	–	–	–	–	–
Smoker (ref: Non-smoker)	–	–	–	–	3.25 (0.07, 6.44)*	1.01	–	–
Mixed (ref: L4/5)	–	–	–7.72 (–13.66, –1.78)*	1.00	–	–	–	–
Accident: Yes (ref: No)	4.39 (0.24, 8.54)*	1.04	–	–	–	–	–	–

Only variables retained in the final stepwise model are shown. Forward stepwise selection (p_entry = 0.05). β = unstandardised regression coefficient. * *p* < 0.05. VIF = variance inflation factor (VIF < 10 = no multicollinearity). Adj. R^2^ = adjusted coefficient of determination. — = variable not retained in model. Abbreviations: DDD, degenerative disc disease; LDH, lumbar disc herniation; PCS, Physical Component Summary; MCS, Mental Component Summary; ODI, Oswestry Disability Index; BMI, body mass index; CI, confidence interval; VIF, variance inflation factor

#### MCS

The MCS models showed limited but variable explanatory power, with adjusted R^2^ values of 0.22 (DDD), 0.15 (spondylolisthesis), 0.14 (stenosis), and 0.09 (LDH) (Table 4). ODI was retained in every MCS model. The stenosis model retained ODI (β − 0.17; 95% CI − 0.21 to − 0.13), Indian ethnicity (β − 4.06; 95% CI − 7.18 to − 0.94), age 65 years and above (β 1.94; 95% CI 0.31 to 3.58), and accident history (β 4.39; 95% CI 0.24 to 8.54). The spondylolisthesis model retained ODI (β − 0.15; 95% CI − 0.21 to − 0.09), Indian ethnicity (β − 6.66; 95% CI − 11.48 to − 1.83), male sex (β 3.11; 95% CI 0.76 to 5.46), and mixed spinal level involvement (β − 7.72; 95% CI − 13.66 to − 1.78). In LDH, ODI (β − 0.13; 95% CI − 0.19 to − 0.08) and current smoker status (β 3.25; 95% CI 0.07 to 6.44) were retained. In the DDD model, ODI (β − 0.17; 95% CI − 0.24 to − 0.10) and pain severity (β − 0.05; 95% CI − 0.10 to − 0.00) were retained.

#### Domain-level regression patterns

Domain-level stepwise regression models (Supplementary Tables S1 and S2) showed a gradient in explanatory power across the eight SF-36 domains. Among the physical domains, PF had the highest adjusted R^2^ of any outcome, including the PCS composite, ranging from 0.57 (spondylolisthesis) to 0.67 (DDD), with ODI retained as the dominant variable in every model. RP showed a similar pattern (adjusted R^2^ 0.34–0.41), with ODI retained as the sole or primary variable. BP models explained 0.26–0.54 of variance, with both ODI and pain severity retained in all four diagnostic groups; ODI had the larger coefficient in three of the four groups. GH models explained smaller proportions of variance (adjusted R^2^ 0.03–0.22).

Among the mental domains, explanatory power varied substantially across domains. SF had the highest adjusted R^2^ among the mental domains (0.31–0.42), followed by VT (0.07–0.28), RE (0.14–0.24), and MH (0.19–0.24), with ODI retained in every model. Indian ethnicity was retained in the RE model for stenosis. The variance explained across all outcomes is summarised in Supplementary Figure S1.

#### Sensitivity analysis

As a sensitivity analysis, post-LASSO OLS models were fitted for all outcomes and diagnostic subgroups (Supplementary Tables S3–S5). The LASSO-selected variables and their coefficients were broadly consistent with the stepwise results, with ODI remaining the dominant predictor of PCS and PF across all diagnostic groups, and MCS models retaining comparable explanatory power (adjusted R^2^ 0.10–0.22). The concordance between the two variable selection approaches supports the robustness of the primary stepwise findings.

## Discussion

Three principal findings emerged: the strong and consistent association between functional disability and physical HRQoL across both composite and domain levels; the limited ability of conventional clinical and demographic variables to explain variance in mental HRQoL; and a gradient in explanatory power across the eight individual SF-36 domains. A fourth observation concerns the comparison across diagnoses: although the four conditions differed statistically on several SF-36 scores, all effect sizes were small, indicating a largely shared preoperative HRQoL burden rather than distinct condition-specific profiles. The clearest, though still modest, separations were a relative preservation of bodily pain in spondylolisthesis and a relative reduction in social functioning in DDD, which may reflect differences in the predominant symptom experience (mechanical or claudicant rather than radicular) across these conditions. This pattern accords with a companion analysis of the same registry using the EQ-5D [22], which found no significant difference in the overall index across the four diagnoses; the more granular SF-36 detected modest domain-level differences, notably in bodily pain and social functioning, yet the overall conclusion of a largely diagnosis-independent preoperative burden was consistent across both instruments and supports standardised, rather than diagnosis-specific, preoperative assessment.

### Functional disability and physical HRQoL

ODI's retention as the strongest PCS predictor in every subgroup (adjusted R^2^ 0.48–0.52) is consistent with the Wilson and Cleary model, in which functional status lies proximal to general health perceptions [10, 11], and mirrors findings from the NORspine registry [16] and the SPORT trial [13]. Interventions directed at functional restoration therefore warrant prioritisation in preoperative care [42, 43]. Notably, pain severity was not retained in any PCS model despite strong univariate associations (β range − 0.13 to − 0.12). This pattern reflects the substantial overlap between pain and functional disability (r 0.53–0.61): once ODI enters the model, it absorbs the shared variance with PCS, rendering pain redundant [44]. This is conceptually expected, as the Wilson and Cleary framework positions symptom status and functional status as closely linked constructs that share considerable explanatory territory [10, 11].

### The opacity of mental HRQoL

A notable finding was the limited ability of the measured variables to explain variance in MCS. With adjusted R^2^ values ranging from 0.09 (LDH) to 0.22 (DDD), the MCS models explained substantially less variance than the PCS models, indicating that the sociodemographic, clinical, and anthropometric characteristics routinely captured in surgical registries had limited ability to account for variation in preoperative mental wellbeing in this population. Within the Wilson and Cleary framework, this pattern is conceptually consistent: overall quality of life and general health perceptions are influenced by individual characteristics, including personality, psychological resilience, coping strategies, and health locus of control [11], which were not measured in this registry. Andersen’s behavioural model offers a similar perspective, suggesting that predisposing factors, particularly psychological dispositions and culturally shaped health beliefs, may have stronger associations with perceived mental wellbeing than need-based clinical factors [12]. The unmeasured psychological factors likely include depression, anxiety, pain catastrophising, self-efficacy, coping style, and health locus of control, while the cultural factors likely include health beliefs, mental health stigma, and culturally patterned idioms of distress and symptom reporting. Future registries could capture these through validated instruments such as the Patient Health Questionnaire-9 (PHQ-9) for depression, the Generalised Anxiety Disorder-7 (GAD-7) scale for anxiety, and the Pain Catastrophising Scale (PCS-13), complemented by brief measures of coping and self-efficacy and by qualitative or mixed-methods enquiry into the culturally shaped beliefs and reporting patterns that quantitative scales may not fully capture.

### Divergences from Western evidence

Several patterns diverged from Western registry evidence. BMI was not retained in any PCS model, despite consistent associations reported in Western cohorts [45]; this may partly reflect the use of Asian-specific thresholds, which classify patients as overweight or obese at lower values and thereby compress between-group differences. Comorbidity was retained only in the DDD PCS model, though the binary granularity of our measurement may have limited detection of dose–response associations. Both findings may reflect Singapore's universally accessible, heavily subsidised healthcare system [46], which supports chronic disease management regardless of BMI or comorbidity burden, potentially attenuating associations observed in settings where access to care is more variable. Ethnicity was largely absent from PCS models (except Malay ethnicity in DDD), suggesting that the physical health constructs measured by the SF-36 perform consistently across ethnic groups, though population-specific norms may be warranted for mental health domains where ethnic variation was observed.

### Counterintuitive associations in MCS models

Several unexpected MCS associations warrant cautious interpretation given limited model fit (adjusted R^2^ 0.09–0.22) and the absence of multiple comparison adjustment. The positive associations between older age and MCS in stenosis, and male sex and MCS in spondylolisthesis, may reflect generational and gender-related differences in psychological adaptation; older adults may have developed greater acceptance of chronic conditions, whilst gender norms in Asian societies may shape the degree to which men report psychological distress [47]. The independent association between Indian ethnicity and lower MCS in both spondylolisthesis and stenosis is novel but should be interpreted cautiously given limited model fit. Within Andersen's framework, ethnicity may act as a predisposing factor embedded within social structure, influencing health beliefs, mental health stigma, and patterns of symptom reporting [48]. Whether this reflects genuine differences in mental wellbeing, differential item functioning of the SF-36 across ethnic groups, or culturally-mediated reporting patterns cannot be determined from these data alone. The positive smoking–MCS association in LDH (β = 3.25, *p* = 0.046) is less readily explained. Smoking may function as a coping strategy for pain-related distress [49], or a "healthy smoker" selection effect may be operating, whereby patients who continue to smoke represent a subgroup with better baseline mental resilience, confounding the association in a cross-sectional design. Neither hypothesis can be evaluated from these data. The positive accident history–MCS association in stenosis was based on only 19 patients (4.0%) and, given the wide confidence interval, is likely a chance finding.

### Insights from domain-level analyses

PF had the highest adjusted R^2^ of any outcome in this study (0.57–0.67), exceeding even the PCS composite, suggesting that the ODI–PCS association is largely concentrated within limitations in physical functioning. Clinically, interventions that improve patients' ability to perform activities of daily living may yield the greatest physical HRQoL gains. BP models retained both ODI and pain severity across all four diagnostic groups, and comorbidity, largely absent from composite PCS models, was consistently retained in general health models, indicating that its influence on HRQoL operates through perceived overall health rather than through physical functioning or pain.

A notable methodological observation emerged from the PF domain models. In the stenosis and spondylolisthesis subgroups, pain severity was retained with a positive coefficient (β = 0.03, *p* = 0.007 and β = 0.05, *p* < 0.05, respectively), reversing its univariate direction of association. This is consistent with a cooperative suppressor effect [50]: once ODI captures the shared variance between pain and physical functioning, the residual variance in pain may index acuity of symptom presentation — that is, patients reporting higher pain relative to their functional limitation may represent a subgroup with better preserved physical functioning. However, coefficient instability due to moderate collinearity between ODI and pain severity (r 0.53–0.61) cannot be excluded, and this finding should be interpreted with caution.

Among the mental domains, SF had the highest adjusted R^2^ (0.31–0.42), with VT (0.07–0.28), RE (0.14–0.24), and MH (0.19–0.24) showing generally lower explanatory power. ODI was retained in every model, suggesting that functional disability is associated with social functioning and emotional role limitations even when its association with overall mental wellbeing (MCS) is weaker. This is consistent with the Wilson and Cleary framework, in which functional status influences specific domains of daily life more directly than it influences global health perceptions [10, 11].

### Strengths and limitations

This study has several strengths: a large registry-based sample spanning four diagnostic conditions, near-complete data (22 exclusions among 1195 enrolled patients), contemporary accrual from 2017 to 2022, multilingual data collection in English and Mandarin, and a multi-ethnic cohort enabling ethnic comparisons not feasible in monoethnic populations.

Several limitations should be acknowledged. First, the cross-sectional design allows identification of associations but does not establish temporal or causal relationships. Second, the single-centre setting and restriction to surgical candidates limit generalisability to the broader population with degenerative lumbar conditions, most of whom are managed conservatively [51]. Third, Chinese ethnic predominance (70.5%) reduced statistical power for analyses within minority subgroups. Fourth, stepwise regression carries recognised risks of model instability and overfitting, although LASSO sensitivity analyses produced concordant results [40]. Fifth, the high ODI–PF association (adjusted R^2^ 0.57–0.67) may partly reflect construct overlap, as both instruments share substantial item content relating to walking, self-care, and standing. Sixth, T-scores were computed using 1998 US population norms to facilitate international comparability; although Singaporean norms have been published [52], US norms were retained because the component summary scores derive from factor coefficients estimated from the US normative sample [28]. Seventh, comorbidity was recorded as a binary variable without granularity regarding the number or type of comorbidities, limiting detection of dose–response associations. Eighth, the registry lacked psychological measures, including depression, anxiety, and pain catastrophising, likely contributing to the limited explanatory power of the MCS models. Finally, no adjustments were made for multiple comparisons, and some associations, particularly in the weakly fitted MCS models, may represent chance findings.

These preoperative scores can also be read against existing Singapore general-population reference values. Thumboo and colleagues established SF-36 version 2 norms for Singapore’s multi-ethnic general population [52]. The substantial physical HRQoL decrement observed here, with mean PCS, physical functioning, and role-physical scores well below the reference mean, is evident whether US or Singaporean general-population values are used as the comparator, indicating that the conclusion of marked preoperative physical burden does not depend on the choice of norm. The present study extends that earlier work in three ways: it moves from general-population description to a surgical clinical population; it provides diagnosis-stratified preoperative SF-36 reference values across four degenerative lumbar conditions rather than a single population estimate; and it identifies the demographic, clinical, and functional factors associated with HRQoL within each condition, thereby supporting clinical interpretation rather than population benchmarking alone.

### Implications and future directions

Several priorities emerge. First, future spine registries should incorporate validated psychological instruments, as the present findings suggest that routine clinical and demographic variables are insufficient to characterise mental HRQoL. Second, preoperative assessment pathways should prioritise functional disability as the factor most strongly associated with physical HRQoL, with implications for prehabilitation programme design and outcome benchmarking. Third, the ethnic variation in mental HRQoL warrants further investigation through qualitative and mixed-methods research exploring cultural mechanisms that may influence mental wellbeing reporting; in the interim, culturally responsive care could include routine screening for psychological distress in groups reporting lower mental HRQoL, the provision of language-concordant and culturally adapted assessment, explicit attention to mental health stigma, and culturally tailored preoperative counselling. Fourth, the diagnosis-specific preoperative SF-36 reference values reported here can serve as interpretive benchmarks for clinicians, supporting individual-level interpretation of a patient’s scores against a condition-relevant baseline, the monitoring of treatment outcomes, and patient-centred shared decision-making. Finally, prospective longitudinal studies would strengthen the evidence base: by repeating SF-36 assessment after surgery, such designs could establish the temporal direction of the associations observed here, characterise individual recovery trajectories, quantify the responsiveness of each domain to surgery, and clarify whether the ethnic differences in mental HRQoL persist, attenuate, or widen over time.

## Conclusion

In this multi-ethnic Southeast Asian cohort, functional disability was most strongly associated with physical HRQoL, whereas conventional variables explained limited mental HRQoL variance. Ethnic variation in mental HRQoL, particularly the association between Indian ethnicity and lower MCS, highlights the importance of culturally-informed assessment in diverse populations. Addressing functional impairment preoperatively and incorporating psychological measures into registries are priorities.

## Supplementary Information

Below is the link to the electronic supplementary material.

Supplementary Material 1

## Data Availability

The data that support the findings of this study are available from the corresponding author upon reasonable request.
