# Supplementary material for: Preoperative health-related quality of life across four degenerative lumbar conditions in a multi-ethnic Southeast Asian surgical cohort: a cross-sectional SF-36 analysis
Source: Qual Life Res. 2026 Jul 16;35(9):242. doi: 10.1007/s11136-026-04336-3 (PMC13395981; doi:10.1007/s11136-026-04336-3)
Supplement: Supplementary file 1 — Supplementary Material 1 [file 11136_2026_4336_MOESM1_ESM.docx]

**Supplementary Table S1. Stepwise MLR: Physical Domain Scores (PF, RP, BP, GH) by Diagnosis**

| **Variable** | **Stenosis** | |  | **Spondylolisthesis** | |  | **LDH** | |  | **DDD** | |
| --- | --- | --- | --- | --- | --- | --- | --- | --- | --- | --- | --- |
|  | **β (95% CI)** | **VIF** |  | **β (95% CI)** | **VIF** |  | **β (95% CI)** | **VIF** |  | **β (95% CI)** | **VIF** |
| **Physical Functioning (PF)** |  |  |  |  |  |  |  |  |  |  |  |
|  | *N=479, Adj.R²=0.6018* |  |  | *N=228, Adj.R²=0.5742* |  |  | *N=262, Adj.R²=0.6030* |  |  | *N=202, Adj.R²=0.6743* |  |
| ODI score | –0.41 (–0.45, –0.38)* | 1.54 |  | –0.43 (–0.49, –0.37)* | 1.59 |  | –0.41 (–0.45, –0.37)* | 1.00 |  | –0.37 (–0.41, –0.34)* | 1.05 |
| Pain score | 0.03 (0.01, 0.06)* | 1.45 |  | 0.05 (0.01, 0.09)* | 1.58 |  | – | – |  | – | – |
| College/Diploma (ref: Primary) | 1.62 (0.17, 3.08)* | 1.03 |  | 3.30 (1.22, 5.37)* | 1.05 |  | – | – |  | – | – |
| Employed (ref: Not employed) | 1.44 (0.16, 2.71)* | 1.06 |  | 1.55 (–0.43, 3.53) | 1.23 |  | – | – |  | – | – |
| ≥1 comorbidity (ref: No comorbidity) | – | – |  | –2.35 (–4.50, –0.21)* | 1.14 |  | – | – |  | –2.32 (–4.06, –0.57)* | 1.04 |
| Overweight (ref: Normal) | – | – |  | – | – |  | – | – |  | 2.17 (0.52, 3.82)* | 1.06 |
| Obese (ref: Normal) | –1.83 (–3.09, –0.56)* | 1.03 |  | – | – |  | – | – |  | – | – |
| Ex-smoker (ref: Non-smoker) | – | – |  | – | – |  | – | – |  | –3.92 (–7.37, –0.46)* | 1.01 |
| L5/S1 (ref: L4/5) | – | – |  | – | – |  | – | – |  | 2.26 (0.18, 4.33)* | 1.04 |
| Others (ref: L4/5) | – | – |  | – | – |  | –3.84 (–7.18, –0.49)* | 1.00 |  | – | – |
| **Role-Physical (RP)** |  |  |  |  |  |  |  |  |  |  |  |
|  | *N=479, Adj.R²=0.3948* |  |  | *N=228, Adj.R²=0.3363* |  |  | *N=262, Adj.R²=0.3601* |  |  | *N=202, Adj.R²=0.4078* |  |
| ODI score | –0.33 (–0.36, –0.29)* | 1.00 |  | –0.32 (–0.38, –0.26)* | 1.00 |  | –0.32 (–0.37, –0.27)* | 1.00 |  | –0.31 (–0.36, –0.26)* | 1.00 |
| Other (ref: Chinese) | –4.27 (–7.39, –1.16)* | 1.00 |  | – | – |  | – | – |  | – | – |
| ≥1 comorbidity (ref: No comorbidity) | – | – |  | – | – |  | – | – |  | –3.07 (–5.46, –0.68)* | 1.00 |
| Others (ref: L4/5) | – | – |  | – | – |  | –4.42 (–8.73, –0.12)* | 1.00 |  | – | – |
| **Bodily Pain (BP)** |  |  |  |  |  |  |  |  |  |  |  |
|  | *N=479, Adj.R²=0.4156* |  |  | *N=228, Adj.R²=0.2646* |  |  | *N=262, Adj.R²=0.3041* |  |  | *N=202, Adj.R²=0.5357* |  |
| ODI score | –0.16 (–0.19, –0.12)* | 1.43 |  | –0.19 (–0.27, –0.11)* | 1.51 |  | –0.16 (–0.21, –0.10)* | 1.58 |  | –0.13 (–0.18, –0.08)* | 1.43 |
| Pain score | –0.12 (–0.15, –0.09)* | 1.43 |  | –0.10 (–0.15, –0.04)* | 1.51 |  | –0.08 (–0.12, –0.04)* | 1.58 |  | –0.15 (–0.18, –0.11)* | 1.44 |
| ≥65 years (ref: 21–44) | – | – |  | – | – |  | – | – |  | 2.78 (0.95, 4.62)* | 1.19 |
| ≥1 comorbidity (ref: No comorbidity) | – | – |  | – | – |  | – | – |  | –2.23 (–4.22, –0.25)* | 1.18 |
| Accident: Yes (ref: No) | – | – |  | – | – |  | – | – |  | –4.26 (–7.96, –0.56)* | 1.02 |
| **General Health (GH)** |  |  |  |  |  |  |  |  |  |  |  |
|  | *N=479, Adj.R²=0.1889* |  |  | *N=228, Adj.R²=0.2051* |  |  | *N=262, Adj.R²=0.0307* |  |  | *N=202, Adj.R²=0.2243* |  |
| ODI score | –0.15 (–0.18, –0.12)* | 1.00 |  | –0.15 (–0.19, –0.10)* | 1.03 |  | –0.05 (–0.09, –0.01)* | 1.00 |  | –0.10 (–0.15, –0.04)* | 1.12 |
| ≥1 comorbidity (ref: No comorbidity) | –2.08 (–3.70, –0.47)* | 1.00 |  | –3.27 (–5.30, –1.25)* | 1.14 |  | –1.96 (–3.80, –0.13)* | 1.00 |  | –4.85 (–7.32, –2.38)* | 1.18 |
| Smoker (ref: Non-smoker) | –2.31 (–4.09, –0.54)* | 1.00 |  | – | – |  | – | – |  | – | – |
| College/Diploma (ref: Primary) | – | – |  | 2.77 (0.82, 4.73)* | 1.05 |  | – | – |  | – | – |
| Employed (ref: Not employed) | – | – |  | –2.43 (–4.28, –0.58)* | 1.21 |  | – | – |  | 4.22 (1.71, 6.74)* | 1.48 |
| ≥65 years (ref: 21–44) | – | – |  | – | – |  | – | – |  | 4.99 (2.37, 7.60)* | 1.57 |
| Overweight (ref: Normal) | – | – |  | – | – |  | – | – |  | 2.41 (0.20, 4.61)* | 1.08 |
| L4/S1 (ref: L4/5) | – | – |  | 2.73 (0.19, 5.28)* | 1.02 |  | – | – |  | – | – |
| Only variables retained in the final stepwise model are shown. Forward stepwise selection (p_entry = 0.05). β = unstandardised regression coefficient. * p < 0.05. VIF = variance inflation factor (VIF < 10 = no multicollinearity). Adj. R² = adjusted coefficient of determination. — = variable not retained in model. Abbreviations: DDD, degenerative disc disease; LDH, lumbar disc herniation; ODI, Oswestry Disability Index; CI, confidence interval; VIF, variance inflation factor. | | | | | | | | | | | |

**Supplementary Table S2. Stepwise MLR: Mental Domain Scores (VT, SF, RE, MH) by Diagnosis**

| **Variable** | **Stenosis** | |  | **Spondylolisthesis** | |  | **LDH** | |  | **DDD** | |
| --- | --- | --- | --- | --- | --- | --- | --- | --- | --- | --- | --- |
|  | **β (95% CI)** | **VIF** |  | **β (95% CI)** | **VIF** |  | **β (95% CI)** | **VIF** |  | **β (95% CI)** | **VIF** |
| **Vitality (VT)** |  |  |  |  |  |  |  |  |  |  |  |
|  | *N=479, Adj.R²=0.1826* |  |  | *N=228, Adj.R²=0.2106* |  |  | *N=262, Adj.R²=0.0700* |  |  | *N=202, Adj.R²=0.2845* |  |
| ODI score | –0.14 (–0.17, –0.11)* | 1.01 |  | –0.16 (–0.21, –0.11)* | 1.00 |  | –0.10 (–0.14, –0.05)* | 1.00 |  | –0.17 (–0.21, –0.12)* | 1.05 |
| Male (ref: Female) | – | – |  | 3.37 (1.57, 5.18)* | 1.00 |  | – | – |  | – | – |
| Indian (ref: Chinese) | – | – |  | –4.11 (–7.81, –0.42)* | 1.00 |  | – | – |  | – | – |
| Smoker (ref: Non-smoker) | –1.76 (–3.47, –0.05)* | 1.04 |  | – | – |  | – | – |  | – | – |
| ≥1 comorbidity (ref: No comorbidity) | –2.27 (–3.82, –0.72)* | 1.03 |  | – | – |  | – | – |  | – | – |
| ≥65 years (ref: 21–44) | 1.38 (0.17, 2.59)* | 1.08 |  | – | – |  | – | – |  | – | – |
| Obese (ref: Normal) | – | – |  | – | – |  | – | – |  | –3.04 (–4.90, –1.18)* | 1.05 |
| **Social Functioning (SF)** |  |  |  |  |  |  |  |  |  |  |  |
|  | *N=479, Adj.R²=0.3302* |  |  | *N=228, Adj.R²=0.3099* |  |  | *N=262, Adj.R²=0.3235* |  |  | *N=202, Adj.R²=0.4241* |  |
| ODI score | –0.31 (–0.35, –0.27)* | 1.00 |  | –0.33 (–0.39, –0.26)* | 1.00 |  | –0.31 (–0.36, –0.25)* | 1.00 |  | –0.30 (–0.37, –0.24)* | 1.39 |
| Pain score | – | – |  | – | – |  | – | – |  | –0.05 (–0.09, –0.00)* | 1.39 |
| Indian (ref: Chinese) | –3.86 (–7.02, –0.69)* | 1.00 |  | –6.30 (–11.58, –1.01)* | 1.02 |  | – | – |  | – | – |
| University & above (ref: Primary) | – | – |  | –4.07 (–7.44, –0.70)* | 1.02 |  | – | – |  | – | – |
| Mixed (ref: L4/5) | – | – |  | –7.70 (–14.17, –1.23)* | 1.00 |  | – | – |  | – | – |
| Others (ref: L4/5) | – | – |  | – | – |  | –5.11 (–9.67, –0.54)* | 1.00 |  | – | – |
| Accident: Yes (ref: No) | – | – |  | – | – |  | 5.23 (0.33, 10.12)* | 1.00 |  | – | – |
| **Role-Emotional (RE)** |  |  |  |  |  |  |  |  |  |  |  |
|  | *N=479, Adj.R²=0.1864* |  |  | *N=228, Adj.R²=0.1359* |  |  | *N=262, Adj.R²=0.1768* |  |  | *N=202, Adj.R²=0.2355* |  |
| ODI score | –0.27 (–0.32, –0.21)* | 1.02 |  | –0.23 (–0.32, –0.15)* | 1.00 |  | –0.24 (–0.31, –0.17)* | 1.01 |  | –0.30 (–0.37, –0.22)* | 1.00 |
| Male (ref: Female) | – | – |  | 4.58 (1.34, 7.81)* | 1.00 |  | – | – |  | – | – |
| Indian (ref: Chinese) | –6.28 (–10.40, –2.15)* | 1.00 |  | – | – |  | – | – |  | – | – |
| Smoker (ref: Non-smoker) | – | – |  | – | – |  | 5.59 (1.50, 9.68)* | 1.01 |  | – | – |
| Accident: Yes (ref: No) | 5.62 (0.14, 11.09)* | 1.02 |  | – | – |  | – | – |  | – | – |
| Mixed (ref: L4/5) | –3.58 (–7.02, –0.13)* | 1.01 |  | – | – |  | – | – |  | – | – |
| Others (ref: L4/5) | – | – |  | – | – |  | –6.95 (–12.55, –1.35)* | 1.00 |  | – | – |
| **Mental Health (MH)** |  |  |  |  |  |  |  |  |  |  |  |
|  | *N=479, Adj.R²=0.2068* |  |  | *N=228, Adj.R²=0.1851* |  |  | *N=262, Adj.R²=0.1893* |  |  | *N=202, Adj.R²=0.2438* |  |
| ODI score | –0.18 (–0.21, –0.15)* | 1.00 |  | –0.18 (–0.23, –0.12)* | 1.00 |  | –0.15 (–0.20, –0.11)* | 1.01 |  | –0.21 (–0.26, –0.16)* | 1.00 |
| Smoker (ref: Non-smoker) | –2.21 (–4.11, –0.32)* | 1.00 |  | – | – |  | – | – |  | – | – |
| Indian (ref: Chinese) | – | – |  | –4.20 (–8.36, –0.03)* | 1.00 |  | – | – |  | – | – |
| Mixed (ref: L4/5) | – | – |  | –7.51 (–12.65, –2.37)* | 1.00 |  | – | – |  | – | – |
| Employed (ref: Not employed) | – | – |  | – | – |  | 3.82 (1.38, 6.26)* | 1.01 |  | – | – |
| Accident: Yes (ref: No) | – | – |  | – | – |  | 4.85 (0.79, 8.91)* | 1.00 |  | – | – |
| Only variables retained in the final stepwise model are shown. Forward stepwise selection (p_entry = 0.05). β = unstandardised regression coefficient. * p < 0.05. VIF = variance inflation factor (VIF < 10 = no multicollinearity). Adj. R² = adjusted coefficient of determination. — = variable not retained in model. Abbreviations: DDD, degenerative disc disease; LDH, lumbar disc herniation; ODI, Oswestry Disability Index; CI, confidence interval; VIF, variance inflation factor. | | | | | | | | | | | |

**Supplementary Table S3. Post-LASSO OLS Sensitivity Analysis: PCS and MCS by Diagnosis**

| **Variable** | **Stenosis** |  | **Spondylolisthesis** |  | **LDH** |  | **DDD** |
| --- | --- | --- | --- | --- | --- | --- | --- |
|  | **β (95% CI)** |  | **β (95% CI)** |  | **β (95% CI)** |  | **β (95% CI)** |
| **Physical Component Summary (PCS)** |  |  |  |  |  |  |  |
|  | *N=480, Adj.R²=0.5283* |  | *N=228, Adj.R²=0.5497* |  | *N=262, Adj.R²=0.4777* |  | *N=202, Adj.R²=0.5117* |
| ODI score | –0.29 (–0.32, –0.25)* |  | –0.32 (–0.36, –0.28)* |  | –0.29 (–0.33, –0.25)* |  | –0.25 (–0.30, –0.20)* |
| Pain score | –0.01 (–0.03, 0.01) |  | — |  | — |  | –0.02 (–0.05, 0.02) |
| 45–64 years (ref: 21–44) | 3.19 (0.27, 6.12)* |  | –2.77 (–5.71, 0.18) |  | — |  | — |
| ≥65 years (ref: 21–44) | 3.00 (–0.04, 6.03) |  | –2.76 (–5.77, 0.24) |  | — |  | — |
| Indian (ref: Chinese) | 0.91 (–1.32, 3.14) |  | — |  | — |  | –1.64 (–5.07, 1.80) |
| Malay (ref: Chinese) | 0.36 (–1.48, 2.19) |  | — |  | — |  | –3.32 (–6.18, –0.45)* |
| Other (ref: Chinese) | –1.56 (–3.98, 0.85) |  | — |  | — |  | –1.17 (–3.70, 1.37) |
| Secondary (ref: Primary) | 0.94 (–0.51, 2.39) |  | 0.42 (–1.62, 2.45) |  | — |  | — |
| College/Diploma (ref: Primary) | 1.71 (0.05, 3.36)* |  | 2.95 (0.77, 5.12)* |  | — |  | — |
| University & above (ref: Primary) | 1.26 (–0.63, 3.15) |  | 1.81 (–0.67, 4.30) |  | — |  | — |
| Employed (ref: Not employed) | 1.14 (–0.14, 2.43) |  | — |  | — |  | 1.21 (–0.60, 3.03) |
| Overweight (ref: Normal) | –0.95 (–2.42, 0.52) |  | –3.29 (–5.29, –1.30)* |  | — |  | 2.28 (–0.01, 4.56) |
| Obese (ref: Normal) | –1.45 (–2.97, 0.08) |  | –3.87 (–5.91, –1.83)* |  | — |  | 1.01 (–1.32, 3.33) |
| ≥1 comorbidity (ref: No comorbidity) | — |  | — |  | — |  | –3.18 (–5.12, –1.24)* |
| Ex-smoker (ref: Non-smoker) | –1.28 (–3.15, 0.59) |  | — |  | — |  | –0.23 (–3.94, 3.49) |
| Smoker (ref: Non-smoker) | –1.54 (–3.20, 0.11) |  | — |  | — |  | –2.70 (–5.49, 0.09) |
| L4/S1 (ref: L4/5) | — |  | 2.11 (–0.34, 4.56) |  | — |  | — |
| L5/S1 (ref: L4/5) | — |  | 0.81 (–1.70, 3.32) |  | — |  | — |
| Mixed (ref: L4/5) | — |  | 1.44 (–2.56, 5.43) |  | — |  | — |
| Others (ref: L4/5) | — |  | 1.17 (–0.65, 2.99) |  | — |  | — |
| **Mental Component Summary (MCS)** |  |  |  |  |  |  |  |
|  | *N=479, Adj.R²=0.1577* |  | *N=228, Adj.R²=0.1350* |  | *N=262, Adj.R²=0.0993* |  | *N=202, Adj.R²=0.2248* |
| ODI score | –0.17 (–0.22, –0.12)* |  | –0.16 (–0.22, –0.09)* |  | –0.08 (–0.15, –0.01)* |  | –0.17 (–0.24, –0.10)* |
| Pain score | –0.01 (–0.04, 0.02) |  | — |  | –0.05 (–0.10, –0.01)* |  | –0.05 (–0.10, –0.00)* |
| Male (ref: Female) | — |  | 3.40 (0.86, 5.93)* |  | — |  | 2.05 (–0.28, 4.37) |
| 45–64 years (ref: 21–44) | 0.18 (–4.10, 4.47) |  | — |  | — |  | — |
| ≥65 years (ref: 21–44) | 1.75 (–2.77, 6.26) |  | — |  | — |  | — |
| Indian (ref: Chinese) | –3.65 (–6.87, –0.42)* |  | –5.96 (–10.96, –0.95)* |  | — |  | — |
| Malay (ref: Chinese) | 0.41 (–2.26, 3.09) |  | –0.08 (–4.28, 4.12) |  | — |  | — |
| Other (ref: Chinese) | –0.42 (–3.83, 3.00) |  | 0.02 (–4.99, 5.02) |  | — |  | — |
| Secondary (ref: Primary) | — |  | 1.46 (–1.72, 4.64) |  | 3.35 (–1.46, 8.16) |  | — |
| College/Diploma (ref: Primary) | — |  | 0.07 (–3.49, 3.63) |  | 2.50 (–2.27, 7.28) |  | — |
| University & above (ref: Primary) | — |  | –1.17 (–5.40, 3.06) |  | 2.15 (–2.49, 6.79) |  | — |
| Employed (ref: Not employed) | –1.06 (–2.93, 0.81) |  | –1.50 (–4.43, 1.44) |  | 1.73 (–1.14, 4.60) |  | — |
| Overweight (ref: Normal) | 2.82 (0.69, 4.95)* |  | 1.63 (–1.49, 4.75) |  | 2.07 (–1.00, 5.13) |  | — |
| Obese (ref: Normal) | 3.71 (1.46, 5.96)* |  | 2.52 (–0.79, 5.84) |  | 2.95 (–0.25, 6.16) |  | — |
| ≥1 comorbidity (ref: No comorbidity) | –2.27 (–4.44, –0.09)* |  | 0.83 (–2.14, 3.80) |  | — |  | — |
| Ex-smoker (ref: Non-smoker) | 1.28 (–1.44, 4.00) |  | — |  | 0.26 (–4.04, 4.57) |  | — |
| Smoker (ref: Non-smoker) | –1.11 (–3.53, 1.31) |  | — |  | 2.60 (–0.80, 6.01) |  | — |
| Accident: Yes (ref: No) | 4.46 (0.22, 8.69)* |  | — |  | 2.19 (–2.60, 6.99) |  | — |
| L4/S1 (ref: L4/5) | 2.98 (0.27, 5.69)* |  | –1.02 (–4.87, 2.82) |  | –0.75 (–6.22, 4.73) |  | — |
| L5/S1 (ref: L4/5) | –0.50 (–4.39, 3.39) |  | 0.58 (–3.23, 4.39) |  | –0.02 (–2.72, 2.68) |  | — |
| Mixed (ref: L4/5) | –0.98 (–3.79, 1.84) |  | –7.71 (–13.98, –1.44)* |  | –1.87 (–9.92, 6.19) |  | — |
| Others (ref: L4/5) | 0.82 (–1.10, 2.74) |  | –0.09 (–2.94, 2.77) |  | –4.99 (–9.55, –0.42)* |  | — |
| Sensitivity analysis. Variables selected by LASSO (least absolute shrinkage and selection operator) with 10-fold cross-validation (Stata 18), then refitted with OLS for unbiased β and 95% CI. When LASSO selected any level of a categorical variable, the full factor variable was included in the OLS refit to maintain interpretable reference categories consistent with the primary stepwise analysis. * p < 0.05. Adj. R² = adjusted coefficient of determination from the post-LASSO OLS model. — = variable not selected by LASSO. Abbreviations: DDD, degenerative disc disease; LDH, lumbar disc herniation; ODI, Oswestry Disability Index; CI, confidence interval; LASSO, least absolute shrinkage and selection operator; OLS, ordinary least squares. | | | | | | | |

**Supplementary Table S4. Post-LASSO OLS Sensitivity Analysis: Physical Domains (PF, RP, BP, GH) by Diagnosis**

| **Variable** | **Stenosis** |  | **Spondylolisthesis** |  | **LDH** |  | **DDD** |
| --- | --- | --- | --- | --- | --- | --- | --- |
|  | **β (95% CI)** |  | **β (95% CI)** |  | **β (95% CI)** |  | **β (95% CI)** |
| **Physical Functioning (PF)** |  |  |  |  |  |  |  |
|  | *N=479, Adj.R²=0.6043* |  | *N=228, Adj.R²=0.6063* |  | *N=262, Adj.R²=0.6007* |  | *N=202, Adj.R²=0.6627* |
| ODI score | –0.40 (–0.44, –0.36)* |  | –0.43 (–0.49, –0.37)* |  | –0.41 (–0.45, –0.37)* |  | –0.37 (–0.41, –0.32)* |
| Pain score | 0.03 (0.01, 0.06)* |  | 0.05 (0.01, 0.09)* |  | — |  | — |
| 45–64 years (ref: 21–44) | 0.61 (–2.64, 3.87) |  | –4.92 (–8.19, –1.64)* |  | — |  | 0.60 (–1.97, 3.18) |
| ≥65 years (ref: 21–44) | 0.08 (–3.32, 3.49) |  | –5.90 (–9.35, –2.46)* |  | — |  | –0.70 (–3.81, 2.41) |
| Indian (ref: Chinese) | — |  | — |  | — |  | –0.53 (–3.88, 2.82) |
| Malay (ref: Chinese) | — |  | — |  | — |  | –2.28 (–5.14, 0.58) |
| Other (ref: Chinese) | — |  | — |  | — |  | –0.75 (–3.26, 1.75) |
| Secondary (ref: Primary) | 1.87 (0.25, 3.49)* |  | 2.44 (0.12, 4.76)* |  | — |  | 0.60 (–2.02, 3.22) |
| College/Diploma (ref: Primary) | 2.89 (1.05, 4.73)* |  | 5.06 (2.50, 7.62)* |  | — |  | 1.16 (–1.55, 3.88) |
| University & above (ref: Primary) | 2.09 (0.05, 4.14)* |  | 2.74 (–0.33, 5.81) |  | — |  | 0.26 (–2.86, 3.38) |
| Employed (ref: Not employed) | 1.04 (–0.37, 2.46) |  | 0.30 (–1.92, 2.51) |  | — |  | 0.40 (–1.71, 2.51) |
| Overweight (ref: Normal) | 0.14 (–1.49, 1.77) |  | –1.82 (–4.12, 0.47) |  | — |  | 2.97 (0.66, 5.27)* |
| Obese (ref: Normal) | –1.50 (–3.20, 0.20) |  | –3.06 (–5.42, –0.69)* |  | — |  | 1.00 (–1.30, 3.30) |
| ≥1 comorbidity (ref: No comorbidity) | –1.06 (–2.72, 0.59) |  | –0.92 (–3.12, 1.28) |  | — |  | –2.09 (–4.13, –0.04)* |
| Ex-smoker (ref: Non-smoker) | –0.70 (–2.77, 1.37) |  | — |  | — |  | –3.86 (–7.49, –0.23)* |
| Smoker (ref: Non-smoker) | –1.29 (–3.11, 0.53) |  | — |  | — |  | –0.96 (–3.73, 1.82) |
| L4/S1 (ref: L4/5) | –0.23 (–2.28, 1.83) |  | — |  | 2.21 (–1.96, 6.39) |  | 0.77 (–1.93, 3.48) |
| L5/S1 (ref: L4/5) | –0.55 (–3.51, 2.40) |  | — |  | –0.29 (–2.28, 1.70) |  | 2.08 (–0.43, 4.59) |
| Mixed (ref: L4/5) | –1.56 (–3.72, 0.61) |  | — |  | –0.96 (–7.14, 5.22) |  | 0.82 (–2.59, 4.22) |
| Others (ref: L4/5) | –0.68 (–2.14, 0.78) |  | — |  | –3.86 (–7.35, –0.36)* |  | –0.39 (–2.65, 1.86) |
| **Role-Physical (RP)** |  |  |  |  |  |  |  |
|  | *N=480, Adj.R²=0.3991* |  | *N=228, Adj.R²=0.3363* |  | *N=262, Adj.R²=0.3589* |  | *N=202, Adj.R²=0.4158* |
| ODI score | –0.33 (–0.37, –0.29)* |  | –0.32 (–0.38, –0.26)* |  | –0.32 (–0.37, –0.26)* |  | –0.30 (–0.35, –0.24)* |
| 45–64 years (ref: 21–44) | 4.01 (0.15, 7.88)* |  | — |  | — |  | — |
| ≥65 years (ref: 21–44) | 3.58 (–0.27, 7.43) |  | — |  | — |  | — |
| Indian (ref: Chinese) | –2.07 (–5.01, 0.87) |  | — |  | — |  | –3.58 (–7.86, 0.69) |
| Malay (ref: Chinese) | 1.69 (–0.75, 4.12) |  | — |  | — |  | –2.98 (–6.66, 0.69) |
| Other (ref: Chinese) | –3.84 (–7.00, –0.68)* |  | — |  | — |  | 1.00 (–2.20, 4.21) |
| ≥1 comorbidity (ref: No comorbidity) | — |  | — |  | — |  | –3.41 (–5.84, –0.99)* |
| L4/S1 (ref: L4/5) | — |  | — |  | 0.53 (−4.83, 5.88) |  | — |
| L5/S1 (ref: L4/5) | — |  | — |  | –1.18 (–3.73, 1.37) |  | — |
| Mixed (ref: L4/5) | — |  | — |  | –5.43 (–13.35, 2.50) |  | — |
| Others (ref: L4/5) | — |  | — |  | –5.04 (–9.52, –0.55)* |  | — |
| **Bodily Pain (BP)** |  |  |  |  |  |  |  |
|  | *N=480, Adj.R²=0.4076* |  | *N=228, Adj.R²=0.2837* |  | *N=262, Adj.R²=0.3041* |  | *N=202, Adj.R²=0.5354* |
| ODI score | –0.16 (–0.19, –0.12)* |  | –0.19 (–0.27, –0.11)* |  | –0.16 (–0.21, –0.10)* |  | –0.13 (–0.18, –0.07)* |
| Pain score | –0.12 (–0.14, –0.09)* |  | –0.09 (–0.15, –0.04)* |  | –0.08 (–0.12, –0.04)* |  | –0.14 (–0.18, –0.11)* |
| Male (ref: Female) | — |  | 3.03 (0.28, 5.77)* |  | — |  | 0.52 (–1.32, 2.35) |
| 45–64 years (ref: 21–44) | 3.61 (0.38, 6.84)* |  | –3.06 (–7.86, 1.74) |  | — |  | 1.60 (–1.12, 4.32) |
| ≥65 years (ref: 21–44) | 4.12 (0.95, 7.29)* |  | –1.53 (–6.55, 3.49) |  | — |  | 3.28 (0.13, 6.42)* |
| Indian (ref: Chinese) | — |  | –4.37 (–9.69, 0.95) |  | — |  | –0.76 (–4.28, 2.76) |
| Malay (ref: Chinese) | — |  | 1.03 (–3.44, 5.49) |  | — |  | –0.84 (–3.84, 2.16) |
| Other (ref: Chinese) | — |  | –1.67 (–6.80, 3.45) |  | — |  | –2.74 (–5.38, –0.10)* |
| Secondary (ref: Primary) | — |  | — |  | — |  | –0.82 (–3.58, 1.94) |
| College/Diploma (ref: Primary) | — |  | — |  | — |  | –1.22 (–4.08, 1.64) |
| University & above (ref: Primary) | — |  | — |  | — |  | –0.01 (–3.33, 3.31) |
| Overweight (ref: Normal) | — |  | –2.54 (–5.76, 0.67) |  | — |  | 2.21 (–0.22, 4.64) |
| Obese (ref: Normal) | — |  | –1.57 (–4.96, 1.83) |  | — |  | 0.57 (–1.88, 3.01) |
| ≥1 comorbidity (ref: No comorbidity) | — |  | — |  | — |  | –3.14 (–5.29, –1.00)* |
| Ex-smoker (ref: Non-smoker) | — |  | –2.61 (–7.32, 2.10) |  | — |  | 3.71 (–0.30, 7.73) |
| Smoker (ref: Non-smoker) | — |  | –4.74 (–9.10, –0.39)* |  | — |  | 0.06 (–2.94, 3.06) |
| Accident: Yes (ref: No) | — |  | — |  | — |  | –5.02 (–9.11, –0.92)* |
| L4/S1 (ref: L4/5) | — |  | 1.82 (–2.12, 5.77) |  | — |  | 1.08 (–1.74, 3.90) |
| L5/S1 (ref: L4/5) | — |  | 0.37 (–3.78, 4.51) |  | — |  | 1.07 (–1.58, 3.71) |
| Mixed (ref: L4/5) | — |  | –4.04 (–10.47, 2.39) |  | — |  | 2.22 (–1.37, 5.82) |
| Others (ref: L4/5) | — |  | 1.42 (–1.53, 4.36) |  | — |  | 1.21 (–1.17, 3.59) |
| **General Health (GH)** |  |  |  |  |  |  |  |
|  | *N=479, Adj.R²=0.2038* |  | *N=228, Adj.R²=0.2165* |  | *N=262, Adj.R²=0.0448* |  | *N=202, Adj.R²=0.2184* |
| ODI score | –0.14 (–0.17, –0.11)* |  | –0.15 (–0.20, –0.11)* |  | — |  | –0.10 (–0.16, –0.04)* |
| Male (ref: Female) | 0.62 (–0.79, 2.03) |  | — |  | — |  | — |
| 45–64 years (ref: 21–44) | 1.06 (–2.22, 4.34) |  | –0.35 (–3.78, 3.08) |  | — |  | 0.28 (–3.11, 3.67) |
| ≥65 years (ref: 21–44) | 2.19 (–1.17, 5.55) |  | 0.81 (–2.97, 4.59) |  | — |  | 4.14 (0.05, 8.23)* |
| Indian (ref: Chinese) | 1.87 (–0.65, 4.39) |  | –2.08 (–5.83, 1.66) |  | — |  | 1.56 (–2.83, 5.95) |
| Malay (ref: Chinese) | 1.45 (–0.61, 3.50) |  | 1.57 (–1.61, 4.75) |  | — |  | –2.25 (–5.99, 1.49) |
| Other (ref: Chinese) | 1.66 (–1.03, 4.36) |  | –0.99 (–4.65, 2.67) |  | — |  | 0.61 (–2.69, 3.90) |
| Secondary (ref: Primary) | 2.23 (0.59, 3.87)* |  | 2.40 (0.12, 4.67)* |  | 5.05 (1.45, 8.66)* |  | 0.94 (–2.49, 4.37) |
| College/Diploma (ref: Primary) | 1.57 (–0.31, 3.44) |  | 5.15 (2.61, 7.70)* |  | 5.39 (1.83, 8.94)* |  | 1.21 (–2.36, 4.77) |
| University & above (ref: Primary) | 2.57 (0.40, 4.74)* |  | 4.26 (1.17, 7.35)* |  | 6.68 (3.29, 10.07)* |  | 1.11 (–2.97, 5.19) |
| Employed (ref: Not employed) | — |  | –3.07 (–5.30, –0.85)* |  | — |  | 4.02 (1.26, 6.78)* |
| Overweight (ref: Normal) | –0.47 (–2.13, 1.20) |  | –1.91 (–4.17, 0.34) |  | — |  | 2.10 (–0.92, 5.13) |
| Obese (ref: Normal) | –0.99 (–2.74, 0.75) |  | –1.50 (–3.88, 0.88) |  | — |  | 0.18 (–2.86, 3.21) |
| ≥1 comorbidity (ref: No comorbidity) | –2.29 (–3.97, –0.61)* |  | –3.34 (–5.55, –1.12)* |  | — |  | –5.14 (–7.82, –2.47)* |
| Ex-smoker (ref: Non-smoker) | –1.06 (–3.25, 1.13) |  | –0.20 (–3.34, 2.94) |  | — |  | –0.15 (–5.07, 4.78) |
| Smoker (ref: Non-smoker) | –2.39 (–4.34, –0.43)* |  | 2.18 (–0.74, 5.11) |  | — |  | –3.59 (–7.25, 0.07) |
| Accident: Yes (ref: No) | — |  | 2.08 (–1.44, 5.60) |  | — |  | 3.61 (–1.47, 8.69) |
| L4/S1 (ref: L4/5) | 2.27 (0.17, 4.37)* |  | 3.25 (0.45, 6.04)* |  | — |  | –0.47 (–4.01, 3.07) |
| L5/S1 (ref: L4/5) | 2.03 (–0.96, 5.03) |  | 1.02 (–1.83, 3.87) |  | — |  | –1.82 (–5.11, 1.46) |
| Mixed (ref: L4/5) | 0.63 (–1.56, 2.82) |  | –1.04 (–5.59, 3.51) |  | — |  | 2.72 (–1.76, 7.20) |
| Others (ref: L4/5) | 1.04 (–0.46, 2.54) |  | 1.29 (–0.77, 3.35) |  | — |  | 0.72 (–2.26, 3.70) |
| Sensitivity analysis. Variables selected by LASSO (least absolute shrinkage and selection operator) with 10-fold cross-validation (Stata 18), then refitted with OLS for unbiased β and 95% CI. When LASSO selected any level of a categorical variable, the full factor variable was included in the OLS refit to maintain interpretable reference categories consistent with the primary stepwise analysis. * p < 0.05. Adj. R² = adjusted coefficient of determination from the post-LASSO OLS model. — = variable not selected by LASSO. Abbreviations: DDD, degenerative disc disease; LDH, lumbar disc herniation; ODI, Oswestry Disability Index; CI, confidence interval; LASSO, least absolute shrinkage and selection operator; OLS, ordinary least squares. | | | | | | | |

**Supplementary Table S5. Post-LASSO OLS Sensitivity Analysis: Mental Domains (VT, SF, RE, MH) by Diagnosis**

| **Variable** | **Stenosis** |  | **Spondylolisthesis** |  | **LDH** |  | **DDD** |
| --- | --- | --- | --- | --- | --- | --- | --- |
|  | **β (95% CI)** |  | **β (95% CI)** |  | **β (95% CI)** |  | **β (95% CI)** |
| **Vitality (VT)** |  |  |  |  |  |  |  |
|  | *N=480, Adj.R²=0.1835* |  | *N=228, Adj.R²=0.2120* |  | *N=262, Adj.R²=0.0870* |  | *N=202, Adj.R²=0.3033* |
| ODI score | –0.13 (–0.16, –0.10)* |  | –0.16 (–0.21, –0.11)* |  | –0.07 (–0.13, –0.02)* |  | –0.14 (–0.19, –0.09)* |
| Pain score | — |  | — |  | –0.02 (–0.06, 0.01) |  | –0.03 (–0.06, 0.01) |
| Male (ref: Female) | 0.70 (–0.62, 2.02) |  | 2.25 (0.21, 4.29)* |  | — |  | — |
| 45–64 years (ref: 21–44) | 1.81 (–1.27, 4.89) |  | –2.20 (–5.78, 1.38) |  | — |  | 3.07 (0.58, 5.55)* |
| ≥65 years (ref: 21–44) | 3.07 (–0.04, 6.19) |  | –0.95 (–4.86, 2.96) |  | — |  | 2.23 (–0.30, 4.75) |
| Indian (ref: Chinese) | — |  | –4.65 (–8.58, –0.71)* |  | — |  | –2.08 (–5.65, 1.50) |
| Malay (ref: Chinese) | — |  | –1.91 (–5.11, 1.28) |  | — |  | 0.06 (–2.98, 3.09) |
| Other (ref: Chinese) | — |  | 0.17 (–3.60, 3.93) |  | — |  | 1.43 (–1.26, 4.12) |
| Secondary (ref: Primary) | 0.42 (–1.12, 1.96) |  | — |  | 4.94 (1.19, 8.70)* |  | — |
| College/Diploma (ref: Primary) | –0.08 (–1.84, 1.68) |  | — |  | 3.40 (–0.31, 7.12) |  | — |
| University & above (ref: Primary) | 1.21 (–0.77, 3.19) |  | — |  | 3.92 (0.35, 7.49)* |  | — |
| Overweight (ref: Normal) | 1.58 (0.01, 3.15)* |  | — |  | — |  | –0.68 (–3.12, 1.76) |
| Obese (ref: Normal) | 0.82 (–0.78, 2.42) |  | — |  | — |  | –3.50 (–5.95, –1.05)* |
| ≥1 comorbidity (ref: No comorbidity) | –2.31 (–3.89, –0.73)* |  | 1.53 (–0.77, 3.84) |  | — |  | — |
| Ex-smoker (ref: Non-smoker) | –0.65 (–2.71, 1.41) |  | 2.41 (–1.08, 5.90) |  | — |  | — |
| Smoker (ref: Non-smoker) | –1.72 (–3.55, 0.12) |  | 2.20 (–0.98, 5.39) |  | — |  | — |
| L4/S1 (ref: L4/5) | — |  | 1.22 (–1.68, 4.13) |  | — |  | — |
| L5/S1 (ref: L4/5) | — |  | 2.22 (–0.78, 5.22) |  | — |  | — |
| Mixed (ref: L4/5) | — |  | 0.98 (–3.78, 5.75) |  | — |  | — |
| Others (ref: L4/5) | — |  | 1.14 (–1.04, 3.32) |  | — |  | — |
| **Social Functioning (SF)** |  |  |  |  |  |  |  |
|  | *N=479, Adj.R²=0.3330* |  | *N=228, Adj.R²=0.3036* |  | *N=262, Adj.R²=0.3077* |  | *N=202, Adj.R²=0.4352* |
| ODI score | –0.31 (–0.35, –0.27)* |  | –0.33 (–0.40, –0.26)* |  | –0.28 (–0.35, –0.21)* |  | –0.30 (–0.37, –0.24)* |
| Pain score | — |  | — |  | –0.03 (–0.08, 0.01) |  | –0.04 (–0.08, 0.01) |
| 45–64 years (ref: 21–44) | 2.73 (–1.56, 7.01) |  | — |  | — |  | — |
| ≥65 years (ref: 21–44) | 3.56 (–0.76, 7.89) |  | — |  | — |  | — |
| Indian (ref: Chinese) | –3.17 (–6.46, 0.11) |  | –7.14 (–12.57, –1.71)* |  | — |  | — |
| Malay (ref: Chinese) | 0.22 (–2.47, 2.91) |  | –1.28 (–5.66, 3.09) |  | — |  | — |
| Other (ref: Chinese) | –2.09 (–5.56, 1.38) |  | –3.75 (–9.12, 1.61) |  | — |  | — |
| Secondary (ref: Primary) | — |  | 1.89 (–1.53, 5.32) |  | — |  | — |
| College/Diploma (ref: Primary) | — |  | 1.68 (–2.00, 5.37) |  | — |  | — |
| University & above (ref: Primary) | — |  | –1.66 (–5.98, 2.66) |  | — |  | — |
| ≥1 comorbidity (ref: No comorbidity) | — |  | 2.27 (−0.77, 5.31) |  | — |  | — |
| Ex-smoker (ref: Non-smoker) | 0.53 (–2.23, 3.30) |  | — |  | — |  | 4.02 (–0.92, 8.95) |
| Smoker (ref: Non-smoker) | –1.58 (–4.03, 0.88) |  | — |  | — |  | –3.10 (–6.70, 0.50) |
| L4/S1 (ref: L4/5) | 2.32 (–0.46, 5.10) |  | 1.63 (–2.50, 5.76) |  | — |  | — |
| L5/S1 (ref: L4/5) | –0.36 (–4.34, 3.61) |  | 0.82 (–3.25, 4.88) |  | — |  | — |
| Mixed (ref: L4/5) | –0.06 (–2.93, 2.82) |  | –6.91 (–13.67, –0.15)* |  | — |  | — |
| Others (ref: L4/5) | 0.10 (–1.85, 2.05) |  | 0.02 (–3.08, 3.11) |  | — |  | — |
| **Role-Emotional (RE)** |  |  |  |  |  |  |  |
|  | *N=479, Adj.R²=0.1963* |  | *N=228, Adj.R²=0.1375* |  | *N=262, Adj.R²=0.1881* |  | *N=202, Adj.R²=0.2355* |
| ODI score | –0.26 (–0.32, –0.19)* |  | –0.23 (–0.31, –0.14)* |  | –0.20 (–0.28, –0.11)* |  | –0.30 (–0.37, –0.22)* |
| Pain score | –0.03 (–0.07, 0.02) |  | — |  | –0.04 (–0.10, 0.02) |  | — |
| Male (ref: Female) | — |  | 4.60 (1.34, 7.86)* |  | — |  | — |
| 45–64 years (ref: 21–44) | –2.44 (–8.11, 3.23) |  | — |  | 2.28 (–1.27, 5.83) |  | — |
| ≥65 years (ref: 21–44) | –1.27 (–7.07, 4.53) |  | — |  | –2.68 (–8.68, 3.32) |  | — |
| Indian (ref: Chinese) | –5.86 (–10.17, –1.55)* |  | — |  | –0.29 (–4.93, 4.36) |  | — |
| Malay (ref: Chinese) | 0.53 (–3.05, 4.12) |  | — |  | –3.43 (–8.26, 1.39) |  | — |
| Other (ref: Chinese) | –0.65 (–5.19, 3.89) |  | — |  | –0.54 (–5.45, 4.37) |  | — |
| Overweight (ref: Normal) | 3.24 (0.39, 6.10)* |  | — |  | 4.64 (0.64, 8.65)* |  | — |
| Obese (ref: Normal) | 4.58 (1.56, 7.59)* |  | — |  | 4.62 (0.36, 8.87)* |  | — |
| ≥1 comorbidity (ref: No comorbidity) | –1.82 (–4.74, 1.09) |  | — |  | –1.37 (–4.84, 2.09) |  | — |
| Ex-smoker (ref: Non-smoker) | 2.00 (–1.64, 5.64) |  | — |  | 1.29 (–4.36, 6.94) |  | — |
| Smoker (ref: Non-smoker) | –0.71 (–3.94, 2.53) |  | — |  | 5.97 (1.66, 10.29)* |  | — |
| Accident: Yes (ref: No) | 5.44 (–0.23, 11.12) |  | — |  | –6.27 (–12.41, –0.13)* |  | — |
| L4/S1 (ref: L4/5) | 2.58 (–1.04, 6.21) |  | –3.85 (–9.04, 1.34) |  | 0.63 (–6.39, 7.66) |  | — |
| L5/S1 (ref: L4/5) | –3.00 (–8.20, 2.21) |  | 0.22 (–4.87, 5.31) |  | –0.32 (–3.82, 3.19) |  | — |
| Mixed (ref: L4/5) | –3.66 (–7.43, 0.12) |  | –6.43 (–14.87, 2.00) |  | –3.00 (–13.38, 7.38) |  | — |
| Others (ref: L4/5) | 0.27 (–2.30, 2.85) |  | –0.34 (–4.16, 3.48) |  | –7.26 (–13.16, –1.36)* |  | — |
| **Mental Health (MH)** |  |  |  |  |  |  |  |
|  | *N=479, Adj.R²=0.2211* |  | *N=228, Adj.R²=0.1818* |  | *N=262, Adj.R²=0.1978* |  | *N=202, Adj.R²=0.2517* |
| ODI score | –0.19 (–0.22, –0.15)* |  | –0.18 (–0.24, –0.13)* |  | –0.13 (–0.19, –0.07)* |  | –0.17 (–0.23, –0.11)* |
| Pain score | — |  | — |  | –0.02 (–0.07, 0.02) |  | –0.03 (–0.08, 0.01) |
| Male (ref: Female) | 0.74 (–0.77, 2.25) |  | — |  | — |  | — |
| 45–64 years (ref: 21–44) | 1.49 (–2.02, 4.99) |  | — |  | — |  | — |
| ≥65 years (ref: 21–44) | 2.29 (–1.41, 5.99) |  | — |  | — |  | — |
| Indian (ref: Chinese) | –1.18 (–3.84, 1.49) |  | –4.08 (–8.29, 0.13) |  | — |  | –3.26 (–7.31, 0.79) |
| Malay (ref: Chinese) | 0.91 (–1.27, 3.10) |  | 0.83 (–2.74, 4.39) |  | — |  | 0.27 (–3.18, 3.72) |
| Other (ref: Chinese) | –0.44 (–3.24, 2.36) |  | 0.14 (–3.99, 4.26) |  | — |  | 0.34 (–2.68, 3.36) |
| Secondary (ref: Primary) | — |  | — |  | 2.73 (–1.41, 6.87) |  | — |
| College/Diploma (ref: Primary) | — |  | — |  | 4.33 (0.23, 8.44)* |  | — |
| University & above (ref: Primary) | — |  | — |  | 2.56 (–1.39, 6.51) |  | — |
| Employed (ref: Not employed) | –0.92 (–2.49, 0.64) |  | — |  | 3.79 (1.36, 6.22)* |  | — |
| Overweight (ref: Normal) | 2.09 (0.33, 3.86)* |  | 1.21 (–1.45, 3.87) |  | 1.65 (–0.91, 4.22) |  | — |
| Obese (ref: Normal) | 2.13 (0.28, 3.98)* |  | 2.57 (–0.26, 5.41) |  | 1.58 (–1.14, 4.29) |  | — |
| ≥1 comorbidity (ref: No comorbidity) | –2.32 (–4.10, –0.53)* |  | 1.67 (−0.69, 4.04) |  | — |  | — |
| Ex-smoker (ref: Non-smoker) | 0.29 (–2.05, 2.63) |  | — |  | — |  | — |
| Smoker (ref: Non-smoker) | –1.53 (–3.60, 0.54) |  | — |  | — |  | — |
| Accident: Yes (ref: No) | — |  | — |  | 4.78 (0.67, 8.89)* |  | — |
| L4/S1 (ref: L4/5) | 1.60 (–0.63, 3.83) |  | −0.47 (−3.74, 2.81) |  | — |  | — |
| L5/S1 (ref: L4/5) | 1.26 (–1.93, 4.45) |  | 0.13 (–3.09, 3.36) |  | — |  | — |
| Mixed (ref: L4/5) | 0.54 (–1.78, 2.86) |  | –7.32 (–12.65, –1.98)* |  | — |  | — |
| Others (ref: L4/5) | 0.81 (–0.79, 2.40) |  | 0.01 (–2.44, 2.46) |  | — |  | — |
| Sensitivity analysis. Variables selected by LASSO (least absolute shrinkage and selection operator) with 10-fold cross-validation (Stata 18), then refitted with OLS for unbiased β and 95% CI. When LASSO selected any level of a categorical variable, the full factor variable was included in the OLS refit to maintain interpretable reference categories consistent with the primary stepwise analysis. * p < 0.05. Adj. R² = adjusted coefficient of determination from the post-LASSO OLS model. — = variable not selected by LASSO. Abbreviations: DDD, degenerative disc disease; LDH, lumbar disc herniation; ODI, Oswestry Disability Index; CI, confidence interval; LASSO, least absolute shrinkage and selection operator; OLS, ordinary least squares. | | | | | | | |


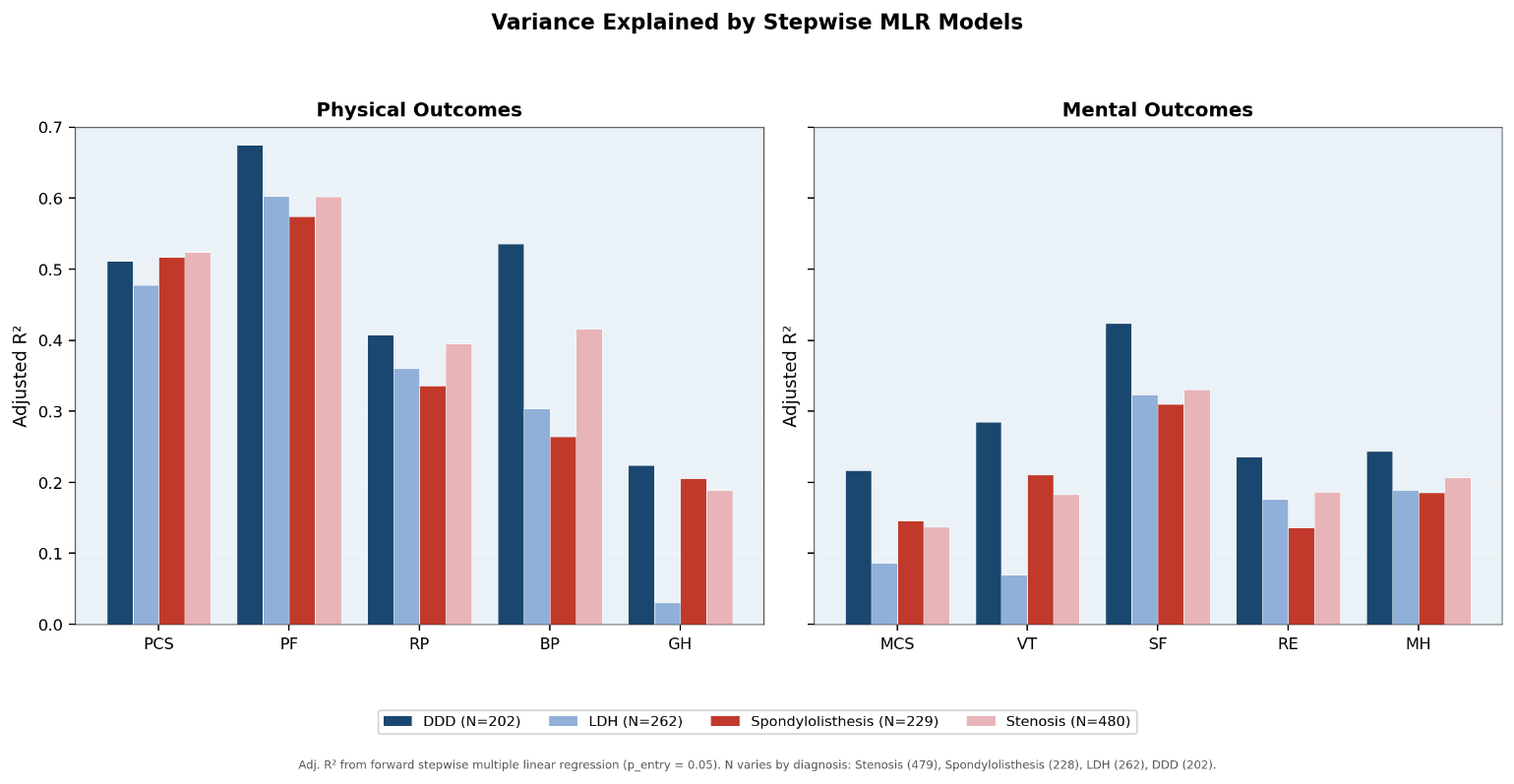


***Supplementary Figure S1. Variance Explained (Adjusted R-squared) Across SF-36 Outcomes by Diagnosis. DDD = degenerative disc disease; LDH = lumbar disc herniation; PCS = Physical Component Summary; MCS = Mental Component Summary; PF = Physical Functioning; RP = Role-Physical; BP = Bodily Pain; GH = General Health; VT = Vitality; SF = Social Functioning; RE = Role-Emotional; MH = Mental Health.***
